# c-Src phosphorylation and activation of hexokinase promotes tumorigenesis and metastasis

**DOI:** 10.1038/ncomms13732

**Published:** 2017-01-05

**Authors:** Jia Zhang, Suili Wang, Bin Jiang, Lihong Huang, Zhiliang Ji, Xiaotong Li, Huamin Zhou, Aidong Han, Ai Chen, Yanan Wu, Huanhuan Ma, Wentao Zhao, Qingwen Zhao, Changchuan Xie, Xiaoyan Sun, Yanming Zhou, Huiying Huang, Muhammad Suleman, Furong Lin, Lin Zhou, Fang Tian, Meijun Jin, Yana Cai, Nan Zhang, Qinxi Li

**Affiliations:** 1 State Key Laboratory of Cellular Stress Biology, Innovation Center for Cell Signaling Network, School of Life Sciences, Xiamen University, Xiamen, Fujian 361102, China; 2 Department of Thoracic Surgery, Chenggong Hospital, Xiamen University, Xiamen, Fujian 361003, China; 3 Department of Hepatobiliary & Pancreatovascular Surgery, The First Affiliated Hospital of Xiamen University, Xiamen, Fujian 361003, China

## Abstract

It is well known that c-Src has important roles in tumorigenesis. However, it remains unclear whether c-Src contributes to metabolic reprogramming. Here we find that c-Src can interact with and phosphorylate hexokinases HK1 and HK2, the rate-limiting enzymes in glycolysis. Tyrosine phosphorylation dramatically increases their catalytic activity and thus enhances glycolysis. Mechanistically, c-Src phosphorylation of HK1 at Tyr732 robustly decreases its *K*_m_ and increases its *V*_max_ by disrupting its dimer formation. Mutation in c-Src phosphorylation site of either HK1 or HK2 remarkably abrogates the stimulating effects of c-Src on glycolysis, cell proliferation, migration, invasion, tumorigenesis and metastasis. Due to its lower *K*_m_ for glucose, HK1 rather than HK2 is required for tumour cell survival when glucose is scarce. Importantly, HK1-Y732 phosphorylation level remarkably correlates with the incidence and metastasis of various clinical cancers and may serve as a marker to predict metastasis risk of primary cancers.

## Abstract (web summary)

The protein tyrosine kinase c-Src is a renowned proto-oncogene with pleiotropic effects. Here, the authors show that c-Src induces the metabolic reprogramming of cancer cells by phosphorylating hexokinases HK1 and HK2, which in turns lead to increased HK catalytic activity and consequent enhancement of glycolysis.

*c-Src*, the counterpart of *v-Src,* is the first proto-oncogene identified in animal cells^1^,^2^. As a tyrosine kinase, c-Src is activated by a variety of cellular factors such as epidermal growth factor (EGF), platelet-derived growth factor (PDGF) and integrin. Active c-Src can phosphorylate various substrates and consequently promote cell survival, proliferation, angiogenesis and motility^3^,^4^. Increased protein levels and/or constitutive activation of c-Src were observed in human cancers originating from a wide spectrum of tissues including colon, breast, lung, liver, pancreas and prostate, implying that uncontrollable activation of c-Src is involved in tumorigenesis and/or metastasis in some of these tumours^3^,^5^.

In recent years, reprogramming of energy metabolism has been considered as an emerging hallmark of cancer^6^. The best-characterized metabolic reprogramming in cancer cells is Warburg effect, which is described as a shift of ATP generation from through oxidative phosphorylation to through glycolysis even under non-hypoxia condition^7^. It was previously reported that a series of recombinant rabbit glycolytic enzymes had been phosphorylated to different extents by pp60 c-Src and pp60 v-Src^8^. *c*-*Src* oncogene could also induce expression of glucose transporter at messenger RNA level^9^. However, up to now it is not yet clear whether c-Src promotes tumorigenesis by directly stimulating Warburg effect. Here we found that c-Src could interact with and phosphorylate human HK1 at Tyr732 and HK2 at Tyr686, which is essential for HK1 and HK2 to catalyse the conversion of glucose to glucose-6-phosphate (G-6-P), the committed step of glycolysis. Substitution of cellular HK1 or HK2 with their corresponding mutants significantly diminishes c-Src stimulated glucose uptake, retarded proliferation and dampened xenograft tumour growth in nude mice.

## Results

### Both HK1 and HK2 interact with c-Src

**Figure 1:**
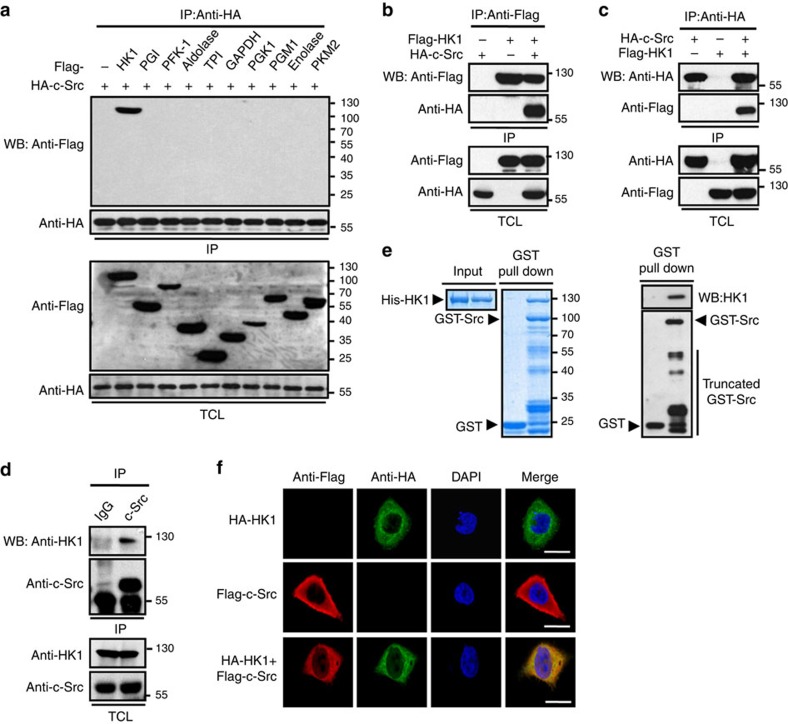
HK1 interacts with c-Src. (**a**) HEK 293T cells were co-transfected with 2 μg of HA-c-Src and equal amount of each of plasmids expressing Flag-tagged enzymes involved in glycolysis (hexokinase 1, HK1; phosphoglucose isomerase, PGI; phosphofructokinase-1, PFK-1; aldolase; triose phosphate isomerase, TPI; glyceraldehydes-3-phosphate dehydrogenase, GAPDH; phosphoglycerate kinase 1, PGK1; phosphoglycerate mutase 1, PGM1; enolase; pyruvate kinase M2, PKM2). Immunoprecipitation (IP) were performed with HA antibody after 24 h of transfection. WB, western blot, TCL, total cell lysate. (**b**,**c**) HEK 293T cells were transfected with HA-c-Src and Flag-HK1 in combinations as indicated. Reciprocal IPs were carried out to precipitate Flag-HK1 (**b**) and HA-c-Src (**c**). (**d**) Endogenous c-Src in lysate of HCT116 cells was precipitated with anti-c-Src, followed by WB to detect c-Src and HK1. (**e**) GST pull down was performed with His-HK1 and GST-c-Src, followed by coomassie brilliant blue staining (left panel) and WB with HK1 antibody for His-HK1 and GST antibody for GST-c-Src. (**f**) HeLa cells were co-transfected with Flag-c-Src and HA-HK1. After 24 h of transfection, immunofluorescence staining was performed to observe the co-localization of c-Src and HK1. Scale bars, 30 μm.

To examine whether c-Src can regulate glycolysis, we performed co-immunoprecipitation (co-IP) assays to seek for any c-Src-interacting proteins involved in glycolysis. Among ten human glycolytic enzymes co-expressed individually with HA-c-Src, HK1 was exclusively precipitated by HA-c-Src (Fig. 1a). This interaction was confirmed by reciprocal co-IP assays with overexpressed HA-c-Src and Flag-HK1 (Fig. 1b,c) and co-IP assay with endogenous proteins (Fig. 1d). *In vitro* GST-pull down assay also confirmed the direct interaction between His-HK1 and GST-c-Src, as indicated by coomassie brilliant blue staining (Fig. 1e, left panel) and western blot (Fig. 1e, right panel). Domain mapping results revealed that SH2 domain (aa 150–249) of c-Src and N-half of HK1 (aa 1–454) were responsible for their mutual interaction (Supplementary Fig. 1a,b). Interestingly, c-Src activity seems to be essential for its interaction with HK1, because such interaction was remarkably diminished by c-Src inhibitor PP2 (Supplementary Fig. 1c), or by replacement of c-Src with c-Src-KD, a kinase dead form of c-Src (Supplementary Fig. 1d). In contrast, such interaction was markedly enhanced by constitutive activation form of c-Src that contains Y529F mutation (Supplementary Fig. 1d). We also found strong co-localization between c-Src and HK1 in cytosol (Fig. 1f). A previous study indicates that HK1 is partially localized in mitochondria where it functions to block apoptotic signals^10^. This prompted us to further explore whether a part of c-Src and HK1 also show mitochondrial location. HK1-RFP (HK1 was fused to red fluorescence protein), Flag-c-Src and Cox 8a-GFP (Cox8a was fused to green fluorescence protein), were co-expressed in HeLa cells. As shown in Supplementary Fig. 1e, the majority of HK1-RFP and Flag-c-Src were localized in cytoplasm while a minor part of them showed mitochondrial location as indicated by Cox 8a-GFP.

HK1 and HK2 are quite different in tissue distribution, kinetic characteristics and regulatory manners^11^. In recent years, excessive expression of either HK1 or HK2 was found in various malignant tumours^12^,^13^. We wanted to know whether HK2 also interacts with c-Src. Both reciprocal co-IPs (Supplementary Fig. 1f,g) and GST-pull down assay (Supplementary Fig. 1h) revealed strong association between HK2 and c-Src. c-Src activity is also required for its interaction with HK2 (Supplementary Fig. 1i,j), analogous to the case of HK1(Supplementary Fig. 1c,d).

### Both HK1 and HK2 are phosphorylated by c-Src

**Figure 2:**
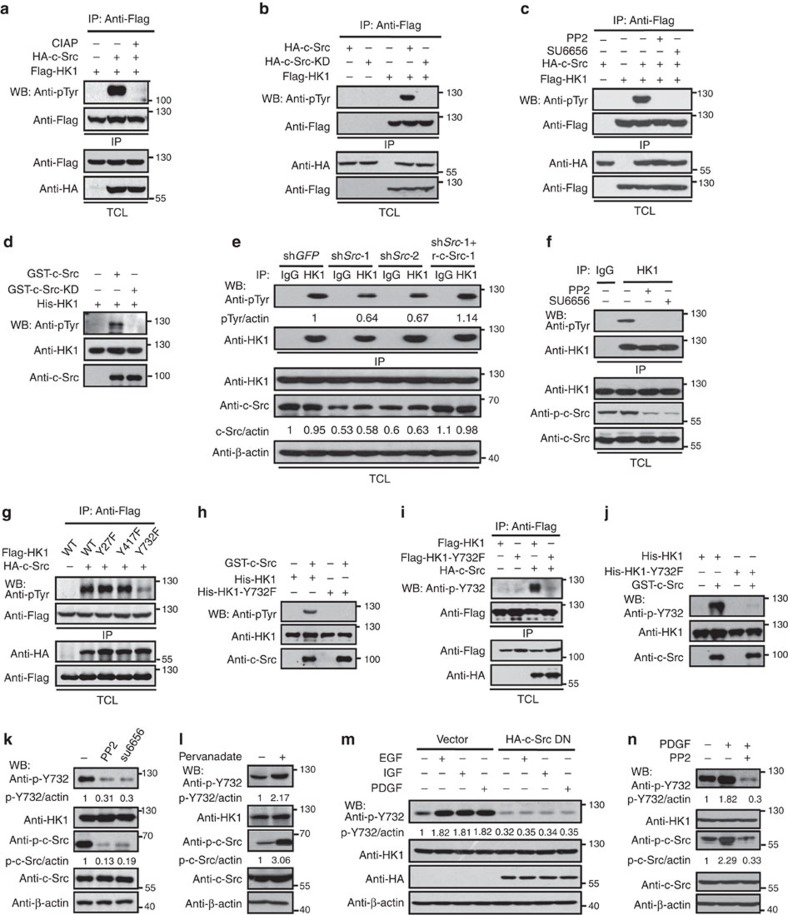
HK1 is phosphorylated by c-Src at Tyr732. (**a**) HEK 293T cells were co-transfected with HA-c-Src and Flag-HK1, followed by IP for HK1 and western blot (WB) with a pan anti-phospho-tyrosine antibody (Anti-pTyr) for detection of HK1 phosphorylation. CIAP, calf-intestinal alkaline phosphatase. (**b**) c-Src, but not c-Src-KD (a kinase dead form of c-Src), is able to phosphorylate HK1 in HEK 293T cells. (**c**) Both PP2 and SU6656, inhibitors of Src family kinases, efficiently antagonize c-Src-mediated phosphorylation of HK1. Transfected HEK 293T cells were treated with or without 10 μM PP2 or 10 μM SU6656 for 4 h before harvest. (**d**) GST-c-Src, but not GST-c-Src-KD, directly phosphorylated His-HK1 in an *in vitro* kinase assay. (**e**) Knockdown of c-Src in HeLa cells impairs tyrosine phosphorylation of HK1. Cells were transfected with pLKO.1-based shRNAs against c-Src or green fluorescence protein (GFP), followed by selection with puromycin (2 μg ml^−1^). Tyrosine phosphorylation of endogenous HK1 was detected. Relative quantification of blots were analysed by Image J software. (**f**) HK1 phosphorylation is abolished by PP2 and SU6656 treatment in HeLa cells. c-Src activity was indicated by detecting Y419 phosphorylation level with anti-p-c-Src. (**g**) Y732F mutation dramatically attenuated c-Src-mediated tyrosine phosphorylation of HK1 in HEK 293T cells. (**h**) HK1-Y732F mutant failed to be phosphorylated by GST-c-Src. *In vitro* kinase assay was performed as in **d**. (**i**) HK1 was phosphorylated by c-Src at Y732 in HEK 293T cells as detected by anti-p-Y732, a home-made rabbit antibody that can specifically recognize phosphorylated Y732 residue. (**j**) His-HK1 is directly phosphorylated by GST-c-Src *in vitro*. (**k**) Inhibition of c-Src activity by PP2 and SU6656 abolishes HK1 phosphorylation at Y732 in HeLa cells. (**l**) HeLa cells were treated with 0.1 mM pervanadate (a protein tyrosine phosphatase inhibitor) for 10 min, followed by detection of HK1-Y732 and c-Src-Y419 phosphorylation. (**m**) Dominant-negative form of c-Src depleted HK1-Y732 phosphorylation stimulated by EGF (100 ng ml^−1^), PDGF (20 ng ml^−1^) or insulin-like growth factor (IGF) (100 ng ml^−1^) in HeLa cells. (**n**) PP2 blocked the phosphorylation of HK1-Y732 stimulated by PDGF in HeLa cells.

Acting as a tyrosine kinase, c-Src regulates cell functions by phosphorylating and activating a variety of its substrate proteins^3^,^4^, therefore, we examined whether c-Src could phosphorylate HK1 and HK2. As shown in Fig. 2a, HA-c-Src could robustly phosphorylate Flag-HK1 and this effect was completely eliminated by calf intestinal alkaline phosphatase. In contrast to wild-type (WT) c-Src, c-Src-KD failed to phosphorylate HK1 (Fig. 2b). This result was confirmed by the observation that c-Src inhibitors PP2 and SU6656 abolished c-Src-induced HK1 phosphorylation (Fig. 2c). Consistently, recombinant His-HK1 was effectively phosphorylated by GST-c-Src, but not by GST-c-Src-KD (Fig. 2d). We also raised two HK1 antibodies by immunizing rabbits with His- or GST-tagged HK1 fragment (aa 316–410). HK1 antibody raised by using GST-tagged fragment showed high potency in performing western blot compared with a commercially available HK1 antibody (Supplementary Fig. 2a) and its specificity was confirmed by using HK1 knocked-down cells (Supplementary Fig. 2b). Immunoprecipitation experiments exhibited that this antibody could also be applied to perform endogenous IP (Supplementary Fig. 2c). Importantly, we detected obvious tyrosine phosphorylation of endogenous HK1 in HeLa cells by using a pan anti-phosphotyrosine antibody, and after knockdown of *c-Src* the phosphorylation was diminished, furthermore, re-expression of rescuing form of c-Src (r-c-Src, expression plasmid of which contains synonymous mutations in short hairpin RNA (shRNA) target sequence and is thus resistant to corresponding shRNA) recovered phosphorylation (Fig. 2e). Consistently, HK1 phosphorylation was also abolished by treatment of cells with either PP2 or SU6656 (Fig. 2f). Similar results were obtained in A549 cells (Supplementary Fig. 2d,e). We also found that HK2 was vigorously phosphorylated by c-Src but not c-Src-KD (Supplementary Fig. 2f), and such an effect of c-Src was abolished by calf intestinal alkaline phosphatase (Supplementary Fig. 2g) or PP2 (Supplementary Fig. 2h). These results were confirmed by *in vitro* kinase assays (Supplementary Fig. 2i). Taken together, our results provide convincing evidence that c-Src is a direct kinase for HK1 and HK2.

We then tried to identify tyrosine residues phosphorylated by c-Src on HK1 and HK2 by employing affinity purification combined with mass spectrometry techniques. Three tyrosine residues of HK1 (Y27, Y417 and Y732) and two residues of HK2 (Y238 and Y686) were identified as candidate phosphorylation sites of c-Src. To confirm mass spectrometry result, the tyrosines were mutated to phenylalanines individually, and the phosphorylation effects of c-Src on them were determined. HK1-Y732F (Fig. 2g) and HK2-Y686F (Supplementary Fig. 2j) mutations dramatically attenuated c-Src-induced phosphorylation of corresponding WT proteins. These results were confirmed by *in vitro* kinase assays of HK1 (Fig. 2h) and HK2 (Supplementary Fig. 2k). To determine whether endogenous HK1 is phosphorylated by c-Src at Y732, we raised an antibody (denoted as anti-p-Y732) that could specifically recognize phosphorylated Y732 as determined by western blot (Fig. 2i,j and Supplementary Fig. 3a,b) and immunohistochemistry staining (IHC) (Supplementary Fig. 3c). We also raised two antibodies by using synthesized HK2 peptides containing phosphorylated Y686. Unfortunately, these antibodies did not show any specificity towards Y686-phosphorylated HK2 (Supplementary Fig. 3d). Then we detected the phosphorylation status of HK1-Y732 in HeLa (Fig. 2k) and A549 (Supplementary Fig. 3e) cells by using anti-p-Y732 antibody and found that endogenous HK1 was apparently phosphorylated at Y732 which was abolished by PP2 and SU6656. In contrast, pervanadate, a pan inhibitor of tyrosine phosphatases, dramatically increased phosphorylation levels of HK1-Y732 and c-Src-Y419 (phosphorylation of this site is used to indicate kinase activity of human c-Src) in HeLa (Fig. 2l) and A549 (Supplementary Fig. 3f) cells.

c-Src can be activated by a series of growth factors such as PDGF, EGF and insulin-like growth factor^3^,^4^, therefore, we examined the effect of these growth factors on HK1 phosphorylation. As shown in Fig. 2m, all of these factors could robustly stimulate HK1-Y732 phosphorylation and such effect was depleted by overexpression of dominant-negative form of c-Src (c-Src DN), indicating that growth factor-stimulated HK1 is mediated by c-Src. Consistently, either PDGF treatment (Fig. 2n) or PDGF receptor (PDGFR) overexpression (Supplementary Fig. 3g) could remarkably stimulate the phosphorylation of both c-Src and HK1-Y732, and such effect was abolished by addition of PP2. We also found that PDGF treatment could enhance the interaction of c-Src with HK1 or HK2, and such effect was abolished by PP2 (Supplementary Fig. 3h). This result demonstrates that c-Src activity is required for PDGF enhanced interaction between c-Src and HK. These observations, along with the results in Supplementary Fig. 1c,d,i,j provide a novel link between growth factor and glycolysis, which is mediated by c-Src activation and its subsequent interaction and phosphorylation of HK. Interestingly, significant correlation between phosphorylation levels of HK1 and c-Src was observed in a series of cancerous human cell lines (Supplementary Fig. 3i). We next detected human foreskin fibroblast cells for c-Src and HK1 phosphorylation and found that both c-Src and HK1 were activated in this cell line and such activation was enhanced by treatment of cells with either PDGF or EGF (Supplementary Fig. 3j), indicating that Src-dependent phosphorylation of HK1 may be a common mechanism in all proliferating cells with Src activated. It is well known that some members of c-Src family kinase are functionally redundant in given signalling pathways and context. Therefore, we examined the effect of other four c-Src family kinase, Hck, Lck, Blk and Yes on the phosphorylation of HK1 and HK2 and found that among these members, only Yes showed much weaker ability in phosphorylating HK1 (Supplementary Fig. 3k) and HK2 (Supplementary Fig. 3l) as compared with c-Src.

### Tyrosine phosphorylation is required for HK activities

**Figure 3:**
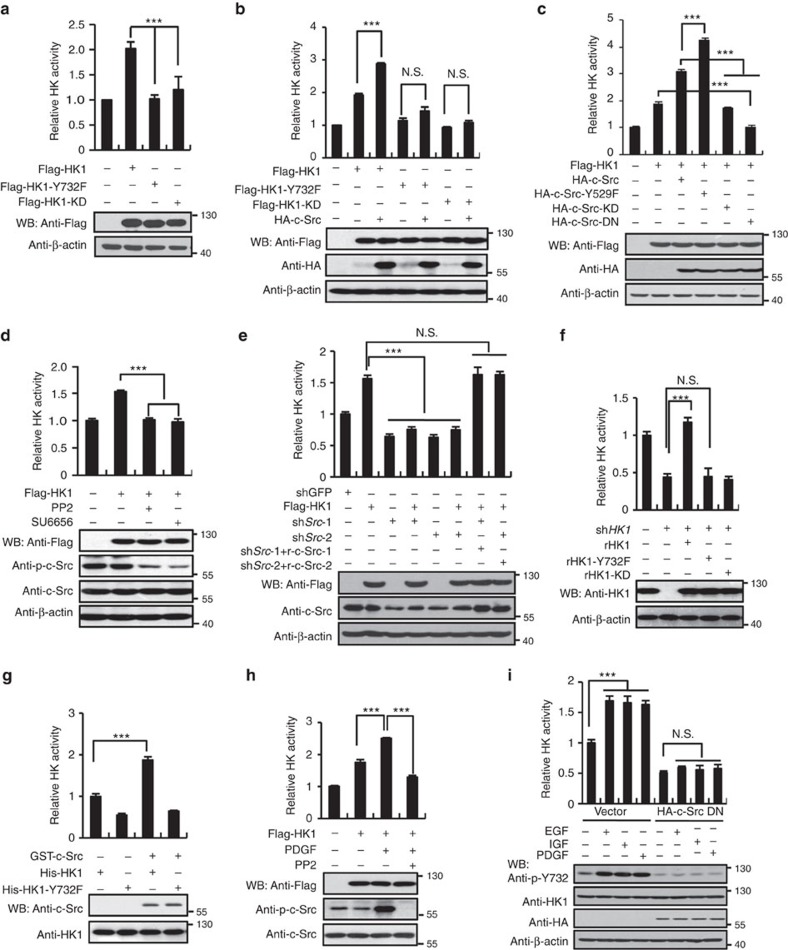
Tyrosine phosphorylation is required for HK activities. (**a**) WT HK1, HK1-Y732F and HK1-KD (a kinase dead form of HK1 containing D657A mutation) were overexpressed in HEK 293T cells, individually, and measured for hexokinase activity according to the protocol described in experimental procedures. (**b**) c-Src robustly promotes kinase activity of WT HK1, but not HK1-Y732F and HK1-KD. HeLa cells were infected with pBOBI-based lentiviruses expressing various proteins indicated. (**c**) c-Src activity regulates the HK1 catalytic potential. HEK 293T cells were co-transfected with different combinations of Flag-HK1, HA-c-Src Y529F (constitutively active form), HA-c-Src KD (kinase dead form K297R) or HA-c-Src DN (dominant-negative form K297R/Y529F). After 24 h, HK1 activity was determined. (**d**) Treatment of HeLa cells with PP2 and SU6656 resulted in inhibition of HK1 activity. (**e**) Knockdown of c-Src dramatically attenuated HK1 activity in HeLa cells, which is reversed by rescue expression of c-Src. Rescue expressions were labelled with a prefix ‘r'. (**f**) In sh*HK1* HeLa cells, HK1 activity was exclusively rescued by re-expressing wild-type HK1. Rescue expressions of WT HK1, HK1-Y732F and HK1-KD were performed based on lentivirus system. (**g**) c-Src stimulates HK1 activity *in vitro*. c-Src kinase assays with HK1 as substrate were performed before HK activity assays. (**h**) PDGF stimulated increase in HK activity was antagonized by PP2 in HeLa cells. (**i**) PDGF, EGF and insulin-like growth factor (IGF) stimulated HK activity to the same extent in HeLa cells and such effect was eliminated by overexpression of dominant-negative form of c-Src. Values in each panel represent means±s.d. of three independent experiments and HK1 activities were normalized to control cells (first bar). Unpaired Student's *t*-test was used to analyse the significance. ***P<0.001, N.S. refers to no significant difference.

We next determined whether tyrosine phosphorylation influences the activities of HK1 and HK2. In comparison with their WT counterparts, both HK1-Y732F (Fig. 3a) and HK2-Y686F (Supplementary Fig. 4a) displayed much lower enzyme activity. Moreover, c-Src could efficiently stimulate the catalytic activities of HK1 and HK2, but failed to stimulate their corresponding mutants (Fig. 3b and Supplementary Fig. 4b). Constitutively activated form of c-Src (c-Src-Y529F) could dramatically activate HK1, while its dominant-negative (DN) form completely abrogated HK1 activity (Fig. 3c). Furthermore, inhibition or knockdown of c-Src abolished HK1 activity in HeLa cells (Fig. 3d,e) and A549 cells (Supplementary Fig. 4c,d). Similar results were observed in the case of HK2 (Supplementary Fig. 4e,f). It is noteworthy that, both two shRNAs employed to knockdown c-Src could function effectively and their influence on HK1 activity was reversed by rescuing expressions of c-Src. We next examined HK activity after knocking down HK1 with shRNA and further rescuing its expression. Re-expression of HK1 rather than HK1-Y732F and HK1-KD, rescued HK activity in HK1-depleted HeLa (Fig. 3f) and A549 cells (Supplementary Fig. 4g). It is the same case to knockdown and re-expression of HK2 and its Y686 mutant (Supplementary Fig. 4h). *In vitro* kinase assay further showed that enzyme activity of WT HK1, rather than HK1-Y732F, had been robustly activated by GST-c-Src (Fig. 3g). In order to exclude the possibility that intrinsic conformation alterations caused by mutations may decrease HK catalytic activity, we carried out partial proteolysis mapping of HK1 and HK2 proteins with trypsin. As shown in Supplementary Fig. 4i,j, the digestion patterns of HK1 and HK2 mutants are the same as their WT controls, indicating that decreased catalytic activity of either HK1-Y732F or HK2 Y686F is not due to conformational alteration caused by Tyr to Phe mutation, but solely due to abolishment of phosphorylation. Moreover, activity of either HK1 or HK2 was effectively promoted by PDGF, and was further antagonized by addition of PP2 (Fig. 3h and Supplementary Fig. 4k). Besides PDGF, EGF and insulin-like growth factor could efficiently stimulate HK activity to the same extent and such effect was abolished by overexpression of c-Src DN (Fig. 3i). Collectively, these observations suggest that c-Src-mediated phosphorylation of HK1 and HK2 is required for their activities promoted by growth factors. We also found that HK1 and HK2 contribute almost equally to total HK activity (Supplementary Fig. 4l).

### c-Src stimulates HK1 by altering Its kinetics

**Figure 4:**
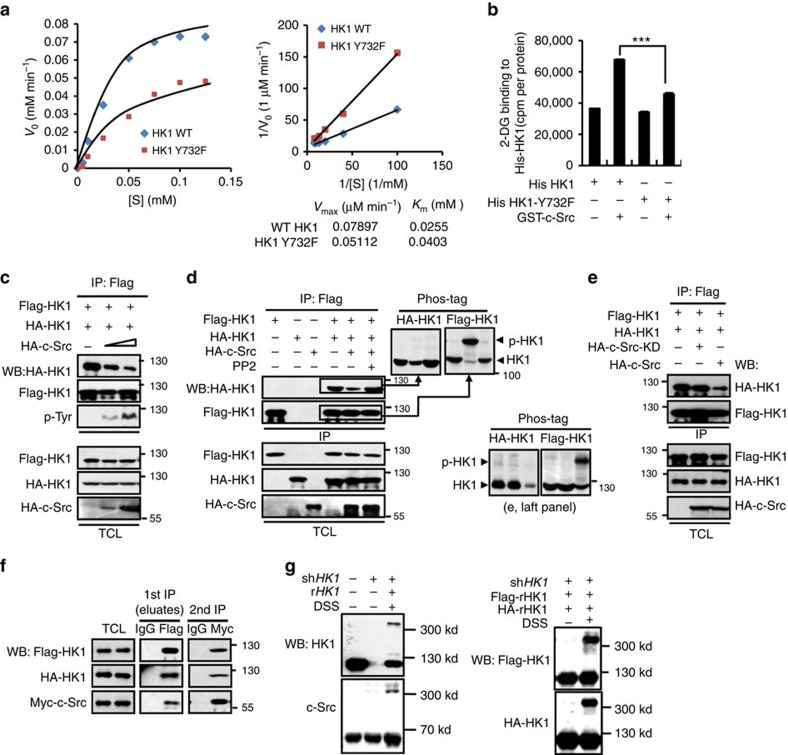
c-Src stimulates HK1 by altering its kinetics. (**a**) Left panel shows the dependence of initial velocities of HK1 and its Y732 mutant on glucose concentrations. Right panel is the double-reciprocal plot of HK1 and HK1-Y732F based on left panel. Experiments were performed in HeLa cells with c-Src highly activated. (**b**) c-Src-mediated phosphorylation of HK1 at Y732 resulted in increased glucose binding to HK1. *In vitro* c-Src kinase assays with HK1 or HK1-Y732 as substrates were carried out before ^3^H 2-DG-binding assays. Values are means±s.d. of three independent experiments. Unpaired Student's *t*-test was used to analyse the *P* value. ***P<0.001. (**c**) c-Src disrupted the formation of HK1 homodimers in a dose-dependent manner. Increasing amounts of c-Src were co-expressed with Flag-HK1 and HA-HK1 in HEK 293T cells, followed by IP and western blot (WB) accordingly. (**d**) c-Src-mediated phosphorylation of HK1 triggered the dissociation of HK1 homodimers and such effect was abolished by treatment with PP2 in HEK 293T cells. The precipitates were isolated by SDS–polyacrylamide gel electrophoresis supplemented with (right panel) or without (left panel) Phostag. (**e**) c-Src-KD was unable to disrupt the formation of HK1 homodimers as compared with its WT counterpart. Left panel shows WB of SDS–polyacrylamide gel electrophoresis with Phostag. (**f**) c-Src forms a ternary complex with HK1 homodimer. Two-step co-IP was performed using HEK 293T cell lysates expressing proteins indicated. First IPs were carried out with IgG (as control) and anti-Flag, followed by elution with the polypeptide of Flag. The eluates were subjected to second IP with control IgG and anti-Myc. (**g**) HeLa cells were lysed in the presence of 5 mM of irreversible chemical crosslinker disuccinimidyl suberate (DSS) at room temperature for 30 min. The reactions were ended with 50 mM Tris (pH 7.5). The cell lysates were then analysed by western blotting.

To explore the mechanism how c-Src-mediated phosphorylation of HK1 and HK2 alters their enzymatic activity, we examined the effect of substrate (glucose) concentration on initial velocity (*V*_0_) of the reaction catalysed by HK1 or its Y732F mutant in the presence of overexpressed c-Src. As shown in Fig. 4a (left panel), Y732F mutation severely diminished the *V*_0_ of HK1-catalysed reaction. We then calculated the maximum velocities (*V*_m_) and Michaelis constants (*K*_m_) of HK1 and HK1-Y732F by making double-reciprocal plots of them. *V*_max_ of HK1-Y732F is obviously lower than that of HK1, however *K*_m_ of this mutant is about two folds that of HK1 (Fig. 4a, right panel), indicating that phosphorylation of Y732 benefits HK1 catalytic efficiency probably by increasing the affinity of enzyme with its substrate glucose. This assumption was confirmed by an *in vitro* experiment showing that c-Src could significantly increase the amount of 2-[1, 2-^3^H]-deoxy-D-glucose associated with HK1 (Fig. 4b).

It was previously reported that HK1 may exist in at least two forms, monomer and dimer^14^,^15^,^16^,^17^. Up to now it is still not clear which form of HK1 is more powerful as a catalyst. We performed co-IP assay with Flag- and HA-tagged HK1 and found that HA-HK1 was effectively precipitated by Flag-HK1, demonstrating that a large part of HK1 exists in the form of homo-dimers (Fig. 4c). When increasing amounts of c-Src were expressed, HA-HK1 precipitated by Flag-HK1 was gradually decreased, negatively correlating with the phosphorylation levels of Flag-HK1 (Fig. 4c), suggesting that c-Src-mediated phosphorylation may lead to the dissociation of HK1 homo-dimer. On the contrary, PP2 treatment relieved the interfering effect of c-Src on HK1 dimer formation (Fig. 4d), however, c-Src-KD failed to show any influence on the formation of this dimer (Fig. 4e). We then isolated phosphorylated- and unphosphorylated-HK1 in the precipitates shown in Fig. 4d,e with phospho-tag gel and detected them with corresponding antibodies (Fig. 4d right panel, and Fig. 4e left panel). In the sample without c-Src overexpression a large amount of unphosphorylated HA-HK1 (lower bands) was co-precipitated by Flag-HK1. Overexpression of c-Src phosphorylated the main part of Flag-HK1 (upper bands), however unphosphorylated HA-HK1 precipitated by Flag-HK1 was dramatically decreased. At the same time, none of phosphorylated HA-HK1 (upper bands) was precipitated by Flag-HK1, demonstrating that Y732 phosphorylation may disrupt the dimerization of HK1.

We next carried out two-step co-IP and found that c-Src could associate with HK1 dimer to form a ternary complex (Fig. 4f). This result was confirmed by western blot based on disuccinimidyl suberate-crosslinking. In HeLa cell lysate cross-linked with disuccinimidyl suberate, we found a protein complex with a molecular weight over 300 kDa that contained HK1 and c-Src (Fig. 4g, left panel). To find out whether this complex contains HK1 dimers, we replaced endogenous HK1 with stably co-expressed HA-HK1 and Flag-HK1, and repeated above experiment. As expected, HA-HK1 and Flag-HK1 were detected in the same protein complex (Fig. 4g, right panel). Because the mass of this protein complex is close to 360 kDa, the molecular weight of an assumed tetramer composed of two molecules of HK1 and two c-Src, we propose that c-Src and HK1 may form tetramer. This proposal needs to be confirmed by further experiments in the future.

### c-Src promotes glycolytic flux by stimulating HK

**Figure 5:**
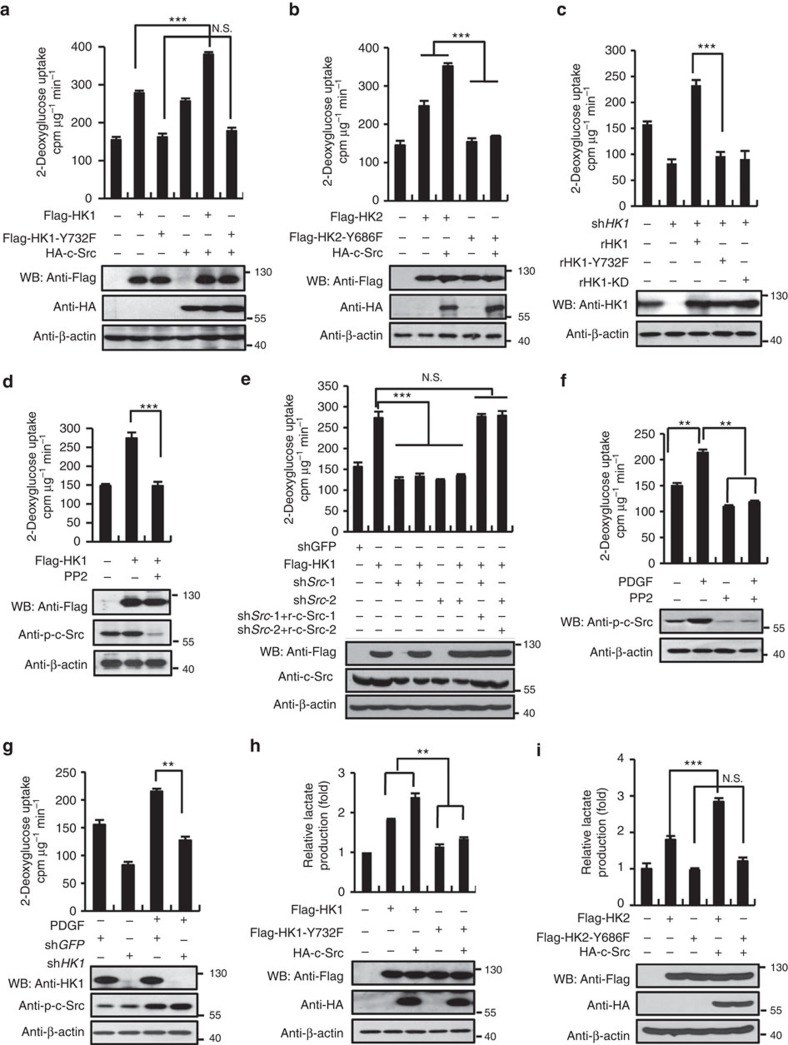
c-Src promotes glycolytic flux by stimulating HK. (**a**) c-Src stimulated glucose uptake by activating HK1 rather than its Y732F mutant. HeLa cells were infected with lentiviruses-expressing proteins indicated. Forty-eight hours post-infection, glucose uptake was measured according to the protocol described in Methods. (**b**) c-Src enhanced glucose uptake promoted by expression of HK2, but not HK2-Y686F in HeLa cells. (**c**) Re-expression of HK1 in HeLa cells rescued the decrease of glucose uptake caused by knockdown of endogenous HK1, while HK1-Y732F and HK1-KD showed no effect. (**d**) The glucose uptake stimulated by HK1 was eliminated by treating HeLa cells with PP2. (**e**) The augmentation of glucose uptake resulted from HK1 overexpression in HeLa cells was diminished by interference of c-Src expression and further rescued by re-expression of c-Src. (**f**,**g**) In HeLa cells PDGF stimulated increase of glucose uptake was diminished by treating cells with PP2 (**f**) or knockdown of HK1 (**g**). (**h**,**i**) Wild-type HK increased the lactate production and this effect was strengthened by co-expression with c-Src. HeLa cells were infected with different combinations of viruses expressing the proteins indicated. Lactate content was detected with lactate assay kit according to the procedure provided in the Methods. Lactate content of each group was normalized to the group of vector control (bar 1). Values in each panel are means±s.d. of three independent experiments. Unpaired Student's *t*-test was used to analyse the significance. **P<0.01, ***P<0.001, N.S. refers to no significant difference.

We then investigated the regulatory effect of c-Src on glycolytic flux by determining 2-[1, 2-^3^H]-deoxy-D-glucose uptake. Overexpressed HK1 (Fig. 5a and Supplementary Fig. 5a) and HK2 (Fig. 5b) significantly stimulated glucose uptake and such effect was enhanced by co-expression of c-Src. In contrast to their WT counterparts, HK1-Y732F and HK2-Y686F failed to increase the efficiency of glucose uptake and also failed to be regulated by c-Src (Fig. 5a,b and Supplementary Fig. 5a). Consistently, re-expression of HK1, but not HK1-Y732F or HK1-KD rescued the decline of glucose uptake caused by knockdown of endogenous HK1 in HeLa (Fig. 5c) and A549 cells (Supplementary Fig. 5b). Furthermore, HK1 stimulated glucose uptake was abolished by PP2 treatment (Fig. 5d and Supplementary Fig. 5c), or knockdown of c-Src (Fig. 5e and Supplementary Fig. 5d). Similar results were obtained to HK2 (Supplementary Fig. 5e,f). PDGF could robustly trigger glucose uptake in both HeLa and A549 cells, and this effect was suppressed either by PP2 treatment (Fig. 5f and Supplementary Fig. 5g) or by knockdown of HK1 (Fig. 5g) or HK2 (Supplementary Fig. 5h), indicating that c-Src activation of HK1 and HK2 is essential for PDGF-stimulated glycolysis. We also examined the effect of other four members of Src family kinase on glucose uptake promoted by overexpression of HK1 or HK2. As expected, all these members showed very weak stimulating effect on glucose flux (Supplementary Fig. 5i,j), consistent with our phosphorylation results (Supplementary Fig. 3k,l).

### c-Src stimulates EMP and PPP flux by activating HK

In tumour cells, elevated glucose consumption is always accompanied with excessive lactate production, we thus detected whether c-Src could also stimulate lactate yield in HeLa cells. As seen in Fig. 5h,i, c-Src could enhance lactate production resulting from overexpression of HK1 and HK2, individually, which is in accordance with glucose uptake results (Fig. 5a,b). Knockdown of HK1 or HK2 decreased lactate production that was rescued by re-expression of their WT forms, rather than their Tyr to Phe mutations (Supplementary Fig. 6a,b).

HK converts glucose to G-6-P that can be either oxidized to pentose via pentose phosphate pathway (PPP) or broken down into pyruvate via glycolysis (also designated as Embden-Meyerhof pathway, EMP). In most types of human cancers, PPP is over-activated^18^,^19^. Additionally, G-6-P dehydrogenase (G6PD) was reported to be activated by c-Src^20^. This promoted us to examine whether c-Src stimulates glucose degradation simultaneously through both EMP and PPP by treating cells with 2-^13^C- glucose and detecting lactate production with NMR (Supplementary Fig. 6c). Overexpressed HK1, but not Y732F increased lactate production from both EMP and PPP, and this ability was strengthened by co-expression of c-Src (Supplementary Fig. 6d, upper panel). Interestingly, although lactate produced in PPP was increased from 0.16 in cells transfected with Flag-HK1 alone to 0.24 in cells co-transfected with Flag-HK1 and HA-c-Src (Supplementary Fig. 6d upper panel), the ratio of lactate yielded in PPP to total lactate production did not show obvious difference between these two groups of cells (9% versus 10%), similar to the case of lactate produced in EMP (Supplementary Fig. 6d, bottom panel). These results indicate that c-Src may promote glucose flux through both EMP and PPP to the same extent simultaneously, and thus not alter the flux ratio of EMP or PPP to total glucose flux. On the contrary, Knockdown of HK1 or replacement of HK1 with its Y732F mutant diminished glucose flux through both EMP and PPP (Supplementary Fig. 6e). As PPP has central role in pentose production essential for nucleic acids biosynthesis and HK1 activity seems to be required for PPP flux, we thus determined DNA/RNA content in HK1 activity deficient cells. As shown in Supplementary Fig. 6f, DNA/RNA synthesis was markedly reduced by knockdown of HK1 or substitution of WT HK1 with its Y732F mutant. Interestingly, HK1 activity deficiency only resulted in mild decline of total intracellular ATP level, but lead to obvious elevation of oxygen consumption while ATP level and oxygen consumption could be severely reduced in HK1 activity deficient cells by inhibition of ATP synthase with oligomycin (Supplementary Fig. 6g,h). These results demonstrate that HK1 deficiency may lead to increased dependence of tumour cells for ATP synthesis based on oxidative phosphorylation.

### c-Src stimulated tumorigenesis depends on both HK1 and HK2

**Figure 6:**
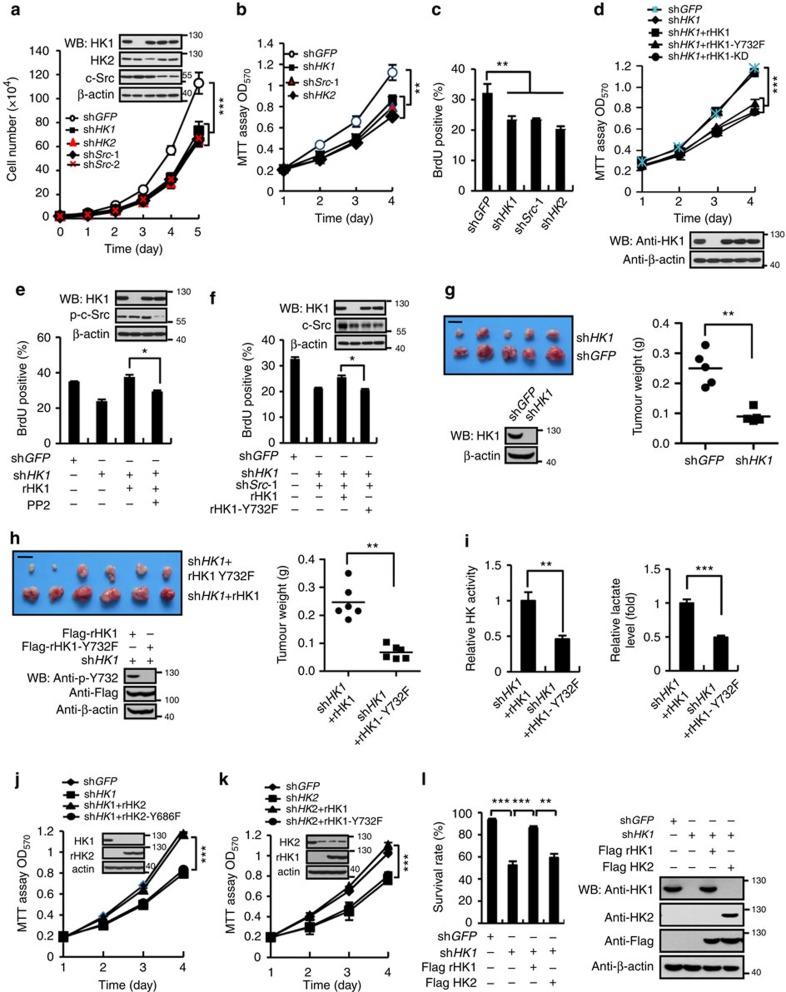
c-Src stimulated tumorigenesis depends on both HK1 and HK2. (**a**–**c**) Individual knockdown of HK1, HK2 and c-Src in HeLa cells resulted in proliferation retardation as determined by growth curves (**a**) MTT assays (**b**) and BrdU incorporation assays (**c**). (**d**) Re-expression of HK1 rescued the decreased proliferation rate in sh*HK1* HeLa cells. (**e**,**f**) The rescued cell proliferation resulted from re-expression of HK1 in sh*HK1* HeLa cells is antagonized by PP2 treatment (**e**) and knockdown c-Src (**f**). (**g**) sh*HK1* significantly attenuated xenograft tumour growth in nude mice. HeLa cells stably expressing sh*HK1* and sh*GFP* were separately injected on the left and right flanks of mouse. Three weeks post-injection, pictures of isolated tumours (scale bars, 6.25 mm) were taken and tumour lysates were detected for HK1 expression (left panel). Tumour weights were determined (*n*=5). (**h**) Replacement of HK1 with HK1-Y732F retarded xenograft tumour growth. sh*HK1* HeLa cells rescued for WT HK1 and HK1-Y732F expressions were injected on the right and left flanks of mice, respectively. Pictures of isolated tumours (scale bars, 6 mm) were taken. Tumours were detected for HK1 expression and HK1-Y732 phosphorylation and tumour weights were determined (*n*=6). (**i**) Tumours shown in **h** were measured for HK activity and lactate production. (**j**) Overexpression of HK2 rescued cell proliferation rate in sh*HK1* HeLa cells. (**k**) Overexpression HK1 rescued the cell proliferation rate in sh*HK2* HeLa cells. (**l**) HK1 was knocked down in A549 cells that are deficient for HK2 expression, followed by rescue expression of HK1 and HK2. Cells were then incubated in medium containing 0.2 mM of glucose for 4 h, followed by determination of survival rates. Western blot (WB) was performed to detect proteins' expressions. The data shown in panels **a**,**c**,**e**,**f**,**i**,**l** are means±s.d. of three independent experiments, in panel **b**,**d**,**j**,**k** are means±s.e.m. of three independent experiments in triplicates. The data in panels **g**,**h** were statistically analysed with two-way ANOVA. Other data were analysed by using unpaired Student's *t*-test. *P<0.05, **P<0.01, ***P<0.001.

We next explored the biological significance underlying c-Src-induced activation of HK1 and HK2. Knockdown of either HK1, HK2 or c-Src dramatically retarded cell proliferation to same extent as indicated by growth curve (Fig. 6a), MTT assays (Fig. 6b) and BrdU incorporation assays (Fig. 6c). Proliferation retardation caused by knockdown of HK1 could be entirely rescued by re-expression of HK1, rather than by HK1-Y732F and HK1-KD (Fig. 6d), however, PP2 treatment abolished such rescuing effect (Fig. 6e). Consistently, in HK1 and c-Src double knockdown cells re-expression of HK1 partially rescued the delayed cell proliferation (Fig. 6f). Knockdown of HK1 significantly slowed down xenograft tumour growth in nude mice (Fig. 6g). Substitution of intracellular HK1 with HK1-Y732F prominently impeded xenograft tumour growth (Fig. 6h) and the tumours containing HK1-Y732F displayed decreased HK activity (Fig. 6i, left panel) and lactate production (Fig. 6i, right panel) versus that expressing WT HK1. Similar result was observed in cells with HK2 replaced with HK2-Y686F (Supplementary Fig. 7a).

We then examined whether HK1 and HK2 distinctly regulate the adaption of cell to different energy status. When cell were cultured in high glucose (25 mM) medium, growth retardation caused by knockdown of HK1 was completely rescued by overexpression of HK2 (Fig. 6j), and vice versa (Fig. 6k), indicating that two isozymes have the same role in cell growth when glucose is plentiful. Glucose deprivation is considered as a feature of solid tumour microenvironment. A previous study shows that colon tumour could retain a mean glucose concentration as low as 0.123 mM (ref. 21). To determine which hexokinase has crucial role in maintaining cell survival when glucose is scarce, we knocked down HK1 in A549 cells (this cell line is abundant of HK1 but absent of HK2) and re-expressed HK1 and HK2, individually. When these cells were exposed to 0.2 mM of glucose, a concentration higher than *K*_m_ of HK1 (0.03 mM), but lower than that of HK2 (0.3 mM) (ref. 11), re-expression of HK1, but not HK2 completely rescued cell survival, indicating that HK1 rather than HK2 sustains the minimum glycolysis for cell survival when glucose concentration is extremely low (Fig. 6l).

### HK activity is required for Src-stimulated tumour metastasis

**Figure 7:**
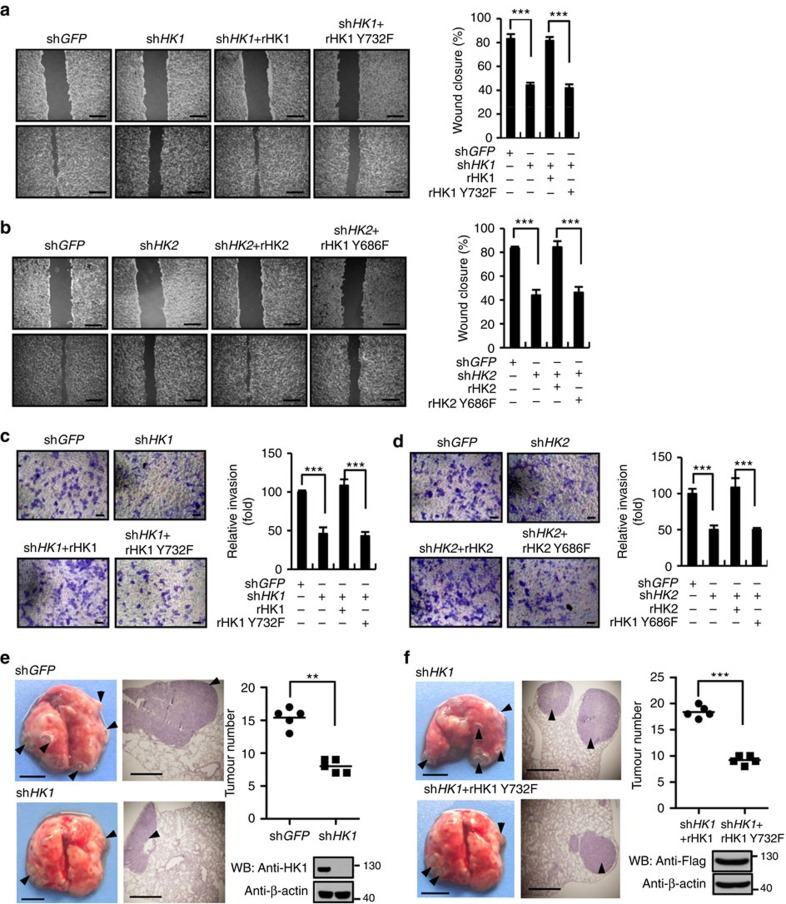
HK activity is required for Src-stimulated tumour metastasis. (**a**,**b**) The wound healing assays show that HK1/HK2 knockdown and rescue expression of their corresponding mutants weakened the migration potential of HCT116 cells. The data are expressed as the means±s.d. of three independent experiments (***P<0.001, unpaired Student's *t*-test). Representative images from one experiment are shown on the left. (**c**,**d**) The transwell assays show that HK1/HK2 knockdown and rescue expression of their corresponding mutants diminished the invasion capability of HCT116 cells. Results are shown as means±s.d. of three independent experiments (***P<0.001, unpaired Student's *t*-test). Representative images from one experiment are shown. (**e**,**f**) The mutation (Y732F) of c-Src phosphorylation site on HK1 disrupted tumour metastasis. *In vivo* metastatic assays were carried out with HCT116 cells according to the protocol shown in Methods. The data in scatter diagrams were analysed by using two-way ANOVA (*n*=5, **P<0.01, ***P<0.001). The scale bars in metastatic lung pictures and haematoxylin and eosin stained tissue picture are 5 and 0.5 mm, respectively.

Excessive expression and/or constitutive activation of c-Src have been well proven to participate in the promotion of cell proliferation, angiogenesis, motility and invasion, and is also involved in metastasis of a wide spectrum of cancers^3^,^4^. It is spontaneous for us to investigate the importance of HK activity in these behaviours of c-Src. In our previous experiments we found that the proteins levels of HK1, HK2 and c-Src are high in HCT116 cells, more importantly, both c-Src and HK1 are robustly activated in this cell line (Supplementary Fig. 3i), we thus used this cell line in the following migration, invasion and metastasis experiments. The results of wound healing assays displayed that HK activity deficiency resulting from knockdown or mutation of HK1 (Fig. 7a) or HK2 (Fig. 7b) remarkably weakened the capabilities of HCT116 cells for migration. Transwell assays indicated that invasion ability of HCT116 cell was remarkably disrupted by knockdown of HK1 or replacement of WT HK1 with its Y732F mutant (Fig. 7c). Similar results were obtained to HK2 (Fig. 7d). These observations demonstrate that phosphorylation of both HK1-Y732 and HK2-Y686 is a crucial step for c-Src to promote cell migration and invasion. We next performed *in vivo* metastatic tumour formation experiments by injecting HCT116 cells into tail veins of nude mice. sh*HK1*-mediated knockdown of HK1 in HCT116 cells significantly decreased the number of metastatic tumour nodules on the surface of lungs, in comparison with control cells expressing sh*GFP* indicating that HK activity is essential for metastatic ability of HCT116 cells (Fig. 7e). Moreover, the impairment of HCT116 cells' metastatic ability caused by knockdown of HK1 could be completely rescued by re-expression of WT HK1, but not HK1 Y732F (Fig. 7f). Tumour nodules were confirmed by haematoxylin and eosin (H&E) staining of the metastasized lungs (Fig. 7e,f). Taken together, these results suggest that HK activity is required for c-Src promoted cell migration and invasion, as well as tumour metastasis.

### HK1-p-Y732 correlates with tumour incidence and metastasis

**Figure 8:**
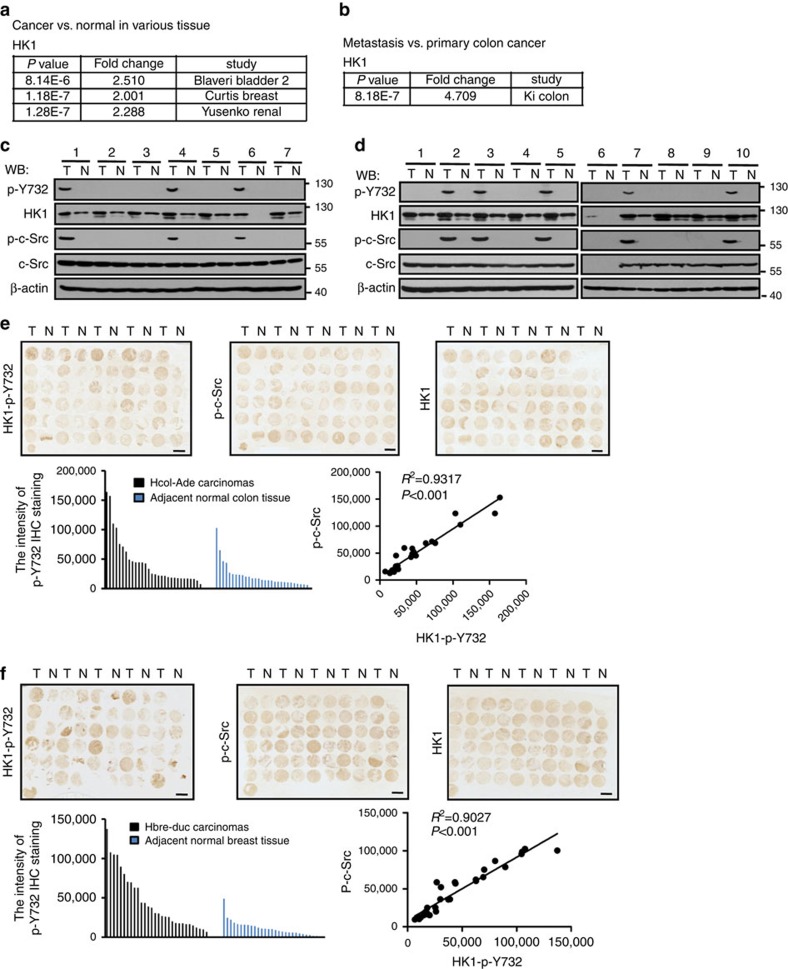
HK1-p-Y732 correlates with tumour incidence and metastasis. (**a**) *P* values and fold changes show increased expression of HK1 in various cancers as compared with normal tissue. Data are publicly available in Oncomine. (**b**) *P* value and fold changes show increased expression of HK1 in metastatic colon cancers compared with primary site tumours. (**c**,**d**) The tumour lysates from patients suffered from lung cancer (**c**) and oesophageal cancer (**d**) were detected with antibodies indicated. T, tumour; N, tumour-adjacent normal tissue. (**e**) IHC staining of successive colon tissue microarrays with HK1-p-Y732 antibody (left), p-c-Src antibody (middle) and HK1 antibody (right). T, Hcol-Ade carcinomas; N, tumour-adjacent normal colon tissues. IPP software was used to evaluate the intensity of the IHC staining. The data were analysed by employing Student's *t*-test. Scale bars, 2 mm. (**f**) IHC staining of the breast tissue microarrays with HK1-p-Y732 antibody (left), p-c-Src antibody (middle) and HK1 antibody (right). T, Hbre-Duc carcinomas; N, tumour-adjacent normal breast tissues. IPP software was used to evaluate the intensity of the IHC staining. The data were analysed by using Student's *t*-test. Scale bars, 2 mm.

After dissecting the significance of HK activity in c-Src promoted tumorigenesis and metastasis, we wanted to know whether there exists any correlation between HK1-Y732 phosphorylation level and tumour incidence or metastasis. Our studies based on Oncomine databases indicate that in bladder cancer, breast cancer and renal cancer, HK1 messenger RNA level is twofold higher than in corresponding normal tissues (Fig. 8a). Moreover, HK1 expression is much higher in metastatic colon cancers than in primary cancers (Fig. 8b). In lung cancer (Fig. 8c), oesophageal cancer (Fig. 8d), breast cancer (Supplementary Fig. 7b) and glioma (Supplementary Fig. 7c), phosphorylation level of HK1-Y732 is perfectly correlated to c-Src activity. To confirm such correlation, we stained successive tissue microarrays of colon adenocarcinoma and breast cancer with HK1-p-Y732, HK1 and p-c-Src antibodies, individually. IHC staining results show that phosphorylation levels of both HK1-Y732 and c-Src in colon adenocarcinoma (Fig. 8e) and breast duct carcinoma (Fig. 8f) are obviously higher than that in corresponding cancer-adjacent normal tissues, meanwhile the intensities of p-c-Src and HK1-p-Y732 are highly correlated with a correlation coefficient of 0.9317 for colon adenocarcinoma and 0.9027 for breast duct carcinoma. In addition, increased phosphorylation of HK1-Y732 was detected in metastatic colon adenocarcinoma compared with primary site tumours (Supplementary Fig. 7d,e). Importantly, phosphorylation level of HK1-Y732 in primary colon adenocarcinoma with metastasis is significantly higher than that in primary colon adenocarcinoma without metastasis (Supplementary Fig. 7f). These results indicate that phosphorylation level of HK1-Y732 is strongly correlated with p-Src and cancer metastasis.

## Discussion

In recent years, both HK1 and HK2 were reported to be highly expressed in malignant tumours^12^,^13^. The correlation between HK2 and tumorigenesis is confirmed by mouse models^22^. However, little is known about the contribution of HK1 to tumorigenesis. Here we found that upon the stimulation of growth factors, such as PDGF, c-Src is activated to promote glycolysis, cell proliferation and tumorigenesis by phosphorylating both HK1 and HK2 and consequently activating their enzymatic activities. Mechanistically, c-Src-mediated Y732 phosphorylation disrupts HK1 dimer formation, alters its enzyme kinetics and eventually enhances enzymatic activity (Supplementary Fig. 7g). Our investigations thus uncover a novel mechanism underlying c-Src stimulated tumorigenesis by promoting Warburg effect.

Interestingly, we found that HK1 and HK2 can distinctively regulate the adaption of tumour cells to different energy status. When glucose concentration is much lower than the *K*_m_ of HK2 (0.3 mM), a scenario frequently occurring in the microenvironment of solid tumour, HK1 acts as the principal HK to provide minimum energy required for cell survival. When glucose concentration is much higher than the *K*_m_ of HK2, two isozymes seems to contribute equally to the total enzymatic activity of hexokinase and cell growth, which is consistent with the previous study^23^. We also found that when intracellular glucose is abundant (25 mM), c-Src stimulates glucose flux through EMP and PPP simultaneously. In this context, HK activity is required for c-Src promoted nucleic acids biosynthesis and proliferation. It was previously reported that in many cancer cells the non-oxidative PPP is activated to produce pentose-5-P (refs 24, 25), this prompt us to determine whether c-Src stimulated HK activity contributes to the activation of this metabolic pathway in future experiments. Taken together, when tumour cells are rich for glucose, both HK1 and HK2 are essential for c-Src stimulated maximum glucose flux required for rapidly proliferating tumour cells.

Our studies based on Oncomine database show that higher expression of HK1 exists in breast cancer, bladder cancer and renal cancer compared with relative normal tissues. We also provide fundamental evidences showing that HK activity is essential for c-Src promoted cell migration, invasion and *in vivo* tumour metastasis. Consistently, clinical investigations also show that HK1-Y732 phosphorylation level is closely correlated with tumour metastasis. Especially, phosphorylation level of HK1-Y732 is markedly higher in primary colon adenocarcinoma with metastasis compared with primary colon adenocarcinoma without metastasis. Taking these lines of evidences into consideration, phosphorylation level of HK1-Y732 may be used as a novel biomarker to predict metastasis risk of primary cancers.

Finally, although inhibitors of hexokinase were proved to be effective in blocking cancer cell proliferation, unacceptable toxicity was observed when these inhibitors are used at high dose^26^. High toxicity of classical hexokinase inhibitor may be due to the possibility that it abolishes glycolysis of normal organs such as brain which mainly depends on glycolysis for energy supply. According to our investigation, small molecules or polypeptides should be developed to disrupt the interaction of c-Src with HK1 and HK2. This kind of inhibitors may show low side effects because they only deprive HKs of excessive activation by c-Src, but keep their basic activity intact.

## Methods

### Constructs

Full length complementary DNA (cDNAs) encoding HK1(gene ID: 3098), HK2 (gene ID: 3099) and c-Src(gene ID: 20779) were obtained from Dr Jiahuai Han (State Key Laboratory of Cellular Stress Biology, School of Life Sciences, Xiamen University) as gifts. Constructs used for transient expression of various proteins were generated based on pCDNA3.3 vectors. Constructs employed for packaging of lentiviruses were created by inserting corresponding cDNAs into pBOBI vectors, individually. Mutations of *HK1, HK2* and *c-Src* were established by a PCR-based site-directed mutagenesis method using Primstar polymerase (Takara). Deletion mutations of *HK1, HK2* and *c-Src* were created by using PCR with their WT cDNA as templates. All PCR products were verified by sequencing (Invitrogen).The details of the primer sequences used for point mutations and deletion mutations are available upon request. pLKO.1-puro lentivirus vector was used to establish the shRNA. The 21-nucleotide sequence for shRNA against human *HK1* is 5′-CACGATGTAGTCACCTTACTA-3′, against *c-Src* is 5′-TCGGCTCATTGAAGACAAT-3′ and 5′-CAGACCTGTCCTTCAAGAA-3′. Additionally, the shRNA sequence targeting human *HK2* is 5′-CTGGCTAACTTCATGGATA-3′.

### Antibodies and reagents

The rabbit polyclonal antibody against HK1 was generated by immunizing rabbits with His-fusion or GST-fusion proteins of human HK1 containing fragments (aa 316–410). The HK1-p-Y732 and HK2-p-Y686 polyclonal antibodies were raised by immunizing rabbits with phospho-peptide VDE{pTyr}SLNAGKQRYEC and GTGSNAC{pTyr}MEEMR, respectively. Mouse anti-HA (clone number F-7, 1:1,000), anti-Myc (clone number 9E10, 1:1,000), anti-c-Src (clone number B-12, 1:1,000) and rabbit anti-HA (clone number Y-11, 1:1,000) were purchased from Santa Cruz. Rabbit anti-HK1 (clone number C35C4, 1:1,000), anti-HK2 (clone number C64G5, 1:1,000), phospho-c-Src (clone number D49G4, 1:1,000) and mouse anti-p-Tyr (catalog number #9411, 1:1,000) were purchased from Cell Signaling Technology. Mouse anti-FLAG (M_2_) (catalog number M8823, 1:5,000), anti-bromodeoxyuridine (clone number BMC9318, 0.2 μg per test) and anti-β-actin (clone number AC-15, 1:5,000) antibodies were purchased from Sigma. Deoxy-D-glucose 2-[1, 2-^3^H] (NET549250UC) and Glucose D-[6-^14^C] (NEC045X050UC) were purchased from PerkinElmer. 2-^13^C-glucose was purchased from Sigma. Various peptides were attained from Gen script (Nanjing) Company.

### Transfection and western blot

Polyethylenimine purchased from Polyscience (23966) was used for transient transfection in HEK 293T cells. To ensure that an equal amount of total DNA was transfected for each sample, corresponding blank vector was used for adjustment of total DNA. To stably reconstruct expression of HK in HeLa cells with its endogenous HK already knocked down by shRNA, we packaged lentiviruses by co-transfecting corresponding pBOBi-based packaging vectors into HEK 293T using Turbofect transfection reagent according to the manufacturer's instruction (#R0532). After purification and titration, a proper amount of virus was used to infect HeLa cells. Cells cultured for immunoprecipitation and western blot were harvested in a lysis buffer (20 mM Tris-HCl, pH 7.4, 150 mM NaCl, 1 mM EDTA, 1 mM EGTA, 1% Triton, 2.5 mM sodium pyrophosphate, 1 mM β-glycerolphosphate, 1 mM sodium orthovanadate, 1 μg ml^−1^ leupeptin, 1 mM phenylmethylsulfonyl fluoride), sonicated fifteen times for one second each, and centrifuged at 15,000*g* for 30 min at 4 °C to obtain supernatant. Immunoprecipitation of target protein was performed by incubating supernatant with corresponding antibody and protein A/G plus-agarose beads (Santa Cruz Biotechnology, Inc.). For western blot, immunoprecipitates or total cell lysates were boiled and separated on 10% SDS–polyacrylamide gels followed by transfering to polyvinylidene difluoride membranes (Roche). After blocking with 5% nonfat milk in Tris-buffered saline containing 0.1% Tween 20 for 1 h, the membranes were probed with corresponding antibodies. Proteins were visualized by enhanced chemiluminescence using horseradish peroxidase-conjugated antibodies. Uncropped scans of the western blots are presented in Supplementary Figures 8-11.

It is worth noting that to detect the endogenous tyrosine phosphorylation of HK1, we harvested 1 × 160 mm dish of HeLa cells with a modified lysis buffer (25 mM Tris-base (pH7.6), 150 mM NaCl, 1% NP-40, 1.5% sodium deoxycholate, 0.1% SDS, 1 mM EDTA, 1 mM Na_3_VO_4_,1 mM PMSF, 5 μg ml^−1^ leupeptin). HK1 was immunoprecipitated with rabbit anti-HK1 antibody (C35C4) followed by detection with mouse anti-p-Tyr (#9411) or home-made rabbit HK1-p-Y732 antibody for HK1 tyrosine phosphorylation.

### Hexokinase activity assay

The activity of hexokinase was tested by employing the method as previously described^23^. In brief, cells cultured in 60 mm dishes were harvested with 400 μl lysis buffer containing 50 mM potassium phosphate, 2 mM dithiothreitol, 2 mM EDTA, and 20 mM sodium fluoride and homogenized at 4 °C by pipetting up and down. The homogenate was centrifuged at 18,000*g* for 15 min at 4 °C and 40 μl of supernatant was added to 1 ml of reaction buffer (100 mM Tris-HCl (pH 8.0), 0.5 mM EDTA, 10 mM ATP, 10 mM MgCl_2_, 2 mM glucose, 0.2 mM NADP^+^ and 30 μg of GST-G6PD purified from *E. coli*). HK activity was determined by following the GST-G6PD dependent conversion of NADP to NADPH spectrophotometrically at 340 nm at 37 °C. The increase in optical density which was measured every 2 min for 15 cycles reflects the increase in NADPH concentration. Total hexokinase activity was calculated according to the slope of the resulting curve in the log phase. HK activities of different samples in the same experiment were normalized to the protein contents in the lysates.

### Identification of HK phosphorylation sites

To map the tyrosine phosphorylation sites of hexokinase which were phosphorylated by c-Src, HEK 293 cells were transfected with Flag-HK1and Flag-HK2 alone or together with HA-c-Src. Twenty-four hours after transfection, Cells were lysed and subjected to immunoprecipitation using Anti-Flag M_2_ beads (Sigma) followed by SDS–polyacrylamide gel electrophoresis. The bands corresponding to HK1 and HK2 were excised from coomassie-stained gels and subjected to digestion. Tryptic peptides were extracted from gels using 0.15% formic acid, 67% acetonitrile and dried for analysis with an AB SCIEX Triple TOF 5600 system.

### Immunofluorescence and immunohistochemistry

HeLa cells cultured on glass coverslips in six-well plates at 30 to 40% of confluence were fixed for 10 min with 100% cold methanol on ice, followed by permeabilization with 0.2% Triton X-100 in phosphate-buffered saline (PBS) for 10 min at room temperature. After washed twice with washing buffer (0.02% Triton X-100, 1.5% BSA and 1 mM NaN_3_ in PBS), the coverslips were blocked with blocking solution (PBS containing 5% BSA, 0.02% Triton X-100 and 1 mM NaN_3_) for 1 h and then incubated with mouse anti-Flag (M2) and rabbit anti-HA antibodies in blocking solution for 1 h. Next, cells were rinsed four times (5 min each) with washing buffer, followed by incubation with appropriate secondary antibodies conjugated with Alexa Fluorescence 488, 594 or 633 (Invitrogen) in blocking solution for 1 h at 37 °C in the dark. Finally the slides were stained with 1 μg ml^−1^ of 4,6-diamidino-2-phenylindole in washing solution for 2 min, washed four times, mounted with 90% glycerol and sealed with nail polish. Images were captured using a Zeiss Laser Scanning Microscope (LSM) 780 at pixels of 1,024 × 1,024.

For immunohistochemistry, 5 μm thick sections were deparaffinized and rehydrated using xylene and a graded series of ethanol (100, 95, 85, 75, 50%), followed by washing in PBS. Antigen retrieval was performed in 10 mM sodium citrate buffer (pH 6.0), which was microwaved at 100 °C for 20 min. After rinsed twice in PBS, sections were blocked at room temperature for 1 h by using 10% normal goat serum, followed by incubation with anti-HK1-p-Y732, anti-c-Src-p and anti-HK1 overnight at 4 °C. Colours were developed with an EliVision super kit (KIT-9921).

### Protein purification and GST-pull down assay

Full length cDNAs of *HK1, HK2* and *SRC* were cloned into pET28a(+) and pGEX 4T-1, individually. Expression plasmids were used for transformation of *E. coli* BL21 strain. Transformed bacteria were induced to express proteins with 0.2 mM isopropyl-β-D-thiogalactoside at 20 °C. Bacteria were harvested until an optical density around 1.0 at 600 nm was reached. His tagged HK1, HK2 and HK1-Y732F or GST-tagged c-Src and c-Src KD were purified with glutathione sepharose beads (GE) or Ni-NTA beads. GST pull down assays were performed by incubating His-HK1 or His-HK2 with GST or GST-c-Src in GST pull-down buffer for 2 h at 4 °C, followed by pulldown with glutathione sepharose beads. The precipitates were washed extensively with above buffer, separated by SDS–polyacrylamide gel electrophoresis and visualized by coomassie brilliant blue staining. Western blot was also carried out using anti-HK1, anti-HK2 and anti-GST antibodies to detect His-HK1, His-HK2 and GST-c-Src present in precipitates, individually.

### *In vitro* kinase assay and glucose-binding assay

As for 2-[1, 2-^3^H]-deoxy-D-glucose binding assay, c-Src kinase assay was firstly performed. After kinase assay, the beads were washed with PBS for three times, followed by incubation with 0.25 μCi 2-[1, 2-^3^H]-deoxy-D-glucose for 30 min at room temperature in kinase assay buffer. The beads were then washed three times with 0.8 ml cold PBS each. Bead-bounded His-HK1 or His-HK1 Y732F proteins were eluted with 30 μl elution buffer (50 mM NaH_2_PO_4_, 0.5 M NaCl, 0.5 M imidazole pH 8.0) for 30 min, followed by detection of radioactivity by virtue of liquid scintillation counter.

### Glucose uptake assay

HEK 293T cells, HeLa cells and A549 cells were plated on six-well culture dishes and cultured with complete medium for 24 h. The complete medium was then replaced by low glucose medium (Hyclone), followed by incubation cells for 4 h. Next, the medium was exchanged for glucose free medium and cells were incubated for another 30 min. Cells were then subjected to 0.25 μCi 2-[1, 2-^3^H]-deoxy-glucose (2-DG, Perkin Elmer) per well. Ten minutes later, cells were washed three times with PBS and lysed in 0.2 M NaOH. Finally, unmetabolized 2-DG in lysate was measured by using liquid scintillation counting.

### BrdU intake assays and MTT assays

Cell proliferation was determined by BrdU intake assay, employing a 5-bromo-2′-deoxyuridine labelling and detection kit (Roche) according to the manufacturer's instructions. In brief, cells were seeded on coverslips in six-well plates. Twenty-four hours later, medium was changed with fresh medium supplemented with BrdU labelling solution. After incubating the cells at 37 °C for 30 min, the BrdU labelling medium was discarded and coverslips were washed three times in washing buffer. Cells were then fixed with 70% ethanol for 30 min at 4 °C, followed by rinsing three times with washing buffer. Fixed cells were incubated with mouse anti-BrdU working solution for 1 h at 37 °C and washed three times. Hereafter, all coverslips were stained with rhodamine-conjugated anti-mouse secondary antibodies for 1 h at 37 °C. After washing three times in wash buffer, coverslips were mounted with 90% glycerol in PBS and kept at 4 °C. Slides were examined under a fluorescence microscope (Olympus IX51) for BrdU staining. To obtain the rate of nuclear BrdU intake, 1,000 cells were counted for each coverslip and the percentage of BrdU-positive nuclei was calculated.

Proliferation rate was also measured by MTT assay. Cells were seeded at a density of 1 × 10^3^ per well in a 96-well plate and grown in complete Dulbecco's modified Eagle's medium (DMEM) medium at 37 °C in 5% CO_2_ for 24 h, followed by incubation with 5 mg ml^−1^ MTT in PBS for another 4 h. Cells were then treated with 150 μl of dissolving reagent dimethylsulphoxide to dissolve the formazan crystals. The optical density was determined using an enzyme-linked immunosorbent assay plate reader with a reference wave length of 570 nm and was normalized by abstracting the value of a blank control.

### Analysis of the PP and EM pathways

The glucose flux through the PPP and EM was estimated with carbon-13 NMR spectrometry. HeLa cells were cultured in 10 cm plates to about 80% confluence. After washed with PBS twice, cells were cultured with medium containing 10 mM [2-^13^C] glucose for 12 h. The medium was collected for analysis of glucose consumption and lactate formation.

### Intracellular ATP assay

ATP concentration was measured with the ATP assay kit (S0026). In brief, cells were seeded in six-well plate for 24 h. Then cells were harvested by using 200 μl lysis buffer and centrifuged at 12,000*g* for 5 min at 4 °C. The supernatant was mixed with detection solution and then analyse for ATP concentration with a luminometer.

### Transwell and wound healing assays

The transwell assay was carried out by using chambers with filters (pore size of 8 μm), coated with Matrigel. HCT116 cell suspensions (1 × 10^5^ cells per well) were seeded into the upper chamber in 200 μl serum-free medium. The lower chamber was applied with complete medium. After incubation for 48 h, invasive cells on the bottom surface of the filters were fixed with 4% formaldehyde for 7 min at room temperature and stained with crystal violet. The cells were counted for five random fields of triplicate replicates. For wound healing assay, HCT116 cells were cultured in 60 mm plate to reach 90% confluence. The cell monolayer was scratched with a pipette tip, washed three times with PBS to remove the detached cells, and incubated in medium containing 10% fetal bovine serum. The scratch areas were photographed at 0 and 48 h. All of the experiments were performed at least three times.

### Lactate production assay

Cellular lactate production was measured with a lactate assay kit (Co-Health (Beijing) Laboratories Co., Ltd., a019-2) under normoxia. In brief, cells were seeded into a six-well plate and cultured for 24 h. The culture medium was changed to fresh medium and incubated for another 4 h. Then, lactate content in cultured medium was determined using the kit.

### Oxygen consumption rate assay

Cells were seeded into the microplates with a density of 5 × 10^3^ cells per plate and cultured overnight. The oxygen consumption rates (OCRs) were measured by Seahorse XF96 instruments (Seahorse Bioscience) after the injection of 0.1 μM oligomycin, 0.05 μM FCCP, 1 μM rotenone and 10 μM atpenin A5. Real-time measurements of oxygen consumption per minute were taken. The mitochondrial oxygen consumption was the difference between basal OCR (before addition of rotenone) and maximal achievable OCR (after addition of rotenone).

### 6-^14^C glucose incorporation into nucleotides

Cells were cultured in 60 mm plates to about 60% confluence. Washed twice with PBS, then the cells were treated with 1 μCi 6-^14^C glucose. After 24 h, cells were harvested. DNA and RNA were isolated with QIAGEN kits according to the instructions and quantified by NanoDrop. Equal volumes of DNA/RNA were added and then radioactivity was measured by liquid scintillation counting and normalized to the DNA/RNA concentration.

### Cell culture

HEK 293T, HeLa, H1299, Hs578T, OVCAR-3, U2OS, A549, SK-MEL-1 and HepG2 were obtained from American Type Culture Collection. HCT116, HT-29, MCF-7, SK-BR-3, MDA-MB-231 and human foreskin fibroblast were purchased from the Cell Bank of the Chinese Academy of Sciences (Shanghai). All cell lines used in this work were maintained in DMEM supplemented with 10% fetal bovine serum and examined negative for mycoplasma contamination using Cycleave PCR Mycoplasma Detection Kit (Takara).

### Patient samples and tissue microarrays

In accordance with research ethics board approval from Xiamen University, Chenggong Hospital of Xiamen University, and The First affiliated Hospital of Xiamen University, primary human breast, lung oesophageal tumour and glioma samples were obtained. Additionally, agreements were received from all patients. The colon and breast tissue chips were purchased from Shanghai Outdo Biotech company and Xian Alenabio company, individually.

### Xenograft tumour and *in vivo* metastasis assays

HeLa cells infected with specific lentiviruses were selected in 2 μg ml^−1^ puromycin for 7 days and subjected to xenograft tumour assays. In all, 1 × 10^7^ cells of individually constructed cell lines were collected and resuspended in DMEM, and then injected subcutaneously into the left or right flank of 6-week-old male BALB/c nude mice purchased from SHANGHAI SLAC LABORATORY ANIMAL COMPANY (*n*≥5). After 3 weeks, the mice were killed, followed by isolation of xenograft tumours whose weights and volumes were determined accordingly.

For *in vivo* metastasis assays, HCT116 cells (1 × 10^6^ in 100 μl PBS ) with HK1 stably knocked down or with endogenous HK1 replaced with rescuing r-HK1 or r-HK1-Y732 were injected into the 4-week-old male BALB/c nude mice via tail veins ((*n*≥5). The mice were killed after 6 weeks and pictures of metastatic lungs were taken. The metastatic tumour nodules on the surface of mouse lungs were counted for statistic analysis. Tumour nodules were confirmed by IHC and western blot.

In all of these animal assays, mice are randomly allocated to the experimental groups. Therefore, the investigators were blinded to the group allocation during the experiment. All animal experimental protocols were approved by the institutional Animal Care and Use Committee at Xiamen University. According to our approved protocol for xenograft tumour assay, tumour size should be inspected daily and not allowed to exceed 1.2 cm in diameter.

### Statistic analyses

Data were represented as means±s.d. or means±s.e.m. of three independent experiments. Animal sample size for each study was selected on the basis of literature documentation of similar well-characterized experiments, and no statistical method was used to predetermine sample size. Statistic analyses were performed by using the two-tailed unpaired Student's *t-*test or two-way ANOVA as indicated in corresponding figure legends. Differences were considered to be statistically significant at *P*<0.05. *P<0.05, **P<0.01, ***P<0.001.

### Data availability

The oncomine data referenced during the study are available in a public repository from the oncomine website (https://www.oncomine.org). The authors declare that all the other data supporting the findings of this study are available within the article and its Supplementary Information Files or from the corresponding author upon reasonable request.

## Additional information

**How to cite this article:** Zhang, J. *et al*. c-Src phosphorylation and activation of hexokinasepromotes tumorigenesis and metastasis. *Nat. Commun.* **8,** 13732 doi: 10.1038/ncomms13732 (2017).

**Publisher's note:** Springer Nature remains neutral with regard to jurisdictional claims in published maps and institutional affiliations.

## Supplementary Material

Supplementary Information Supplementary Figures 1 - 11
