# Supplementary material for: c-Src phosphorylation and activation of hexokinase promotes tumorigenesis and metastasis
Source: Nat Commun. 2017 Jan 5;8:13732. doi: 10.1038/ncomms13732 (PMC5227066; doi:10.1038/ncomms13732)
Supplement: Supplementary Information — Supplementary Figures 1 - 11 [file ncomms13732-s1.pdf]

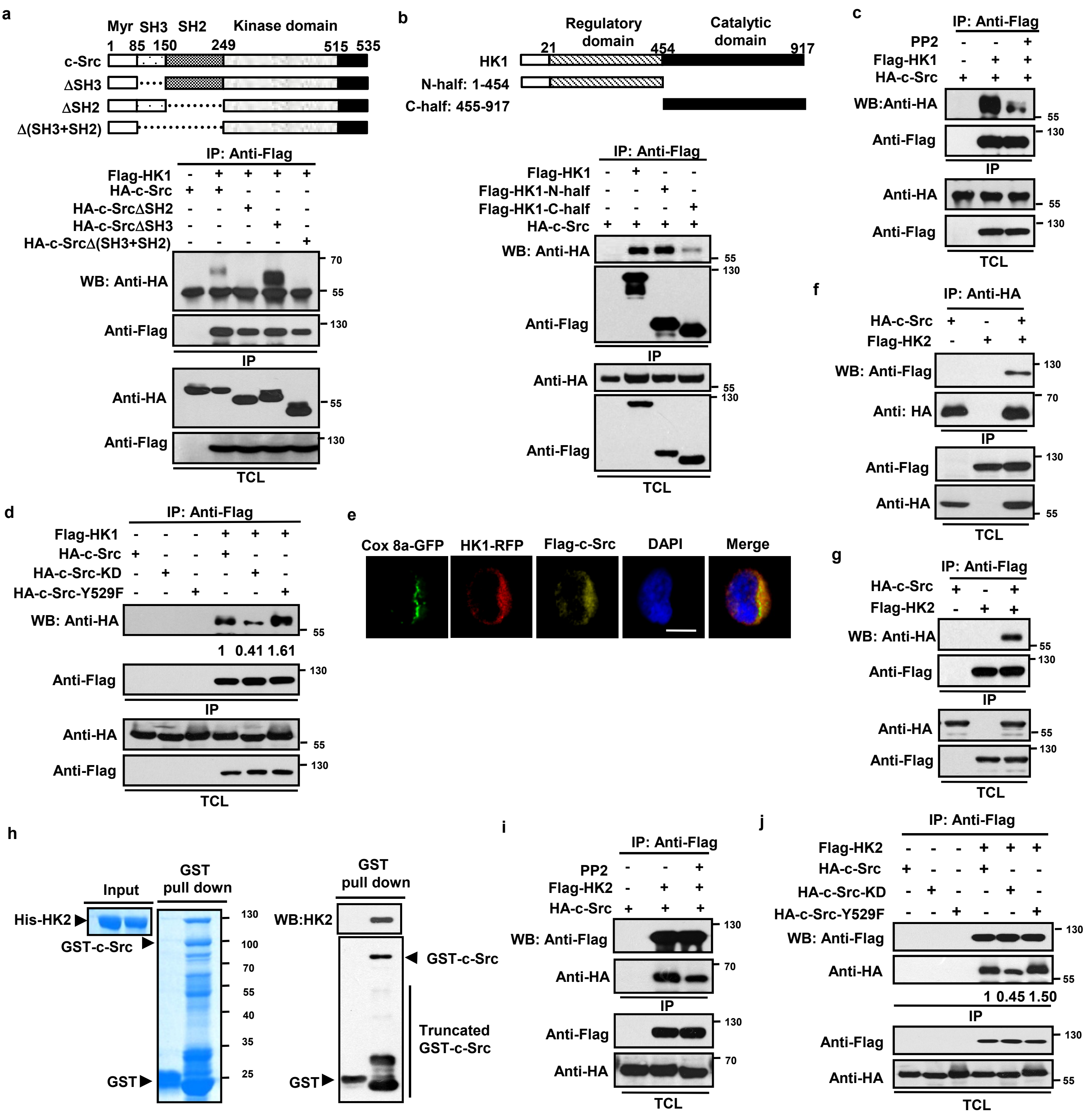

a
Myr SH3 SH2 Kinase domain
1 85 150 249 515 535
c-Src
ΔSH3
ΔSH2
Δ(SH3+SH2)
IP: Anti-Flag
Flag-HK1
HA-c-Src
HA-c-SrcΔSH2
HA-c-SrcΔSH3
HA-c-SrcΔ(SH3+SH2)
WB: Anti-HA
Anti-Flag
IP
Anti-HA
Anti-Flag
TCL
b
Regulatory domain
Catalytic domain
21 454 917
HK1
N-half: 1-454
C-half: 455-917
IP: Anti-Flag
Flag-HK1
Flag-HK1-N-half
Flag-HK1-C-half
HA-c-Src
WB: Anti-HA
Anti-Flag
IP
Anti-HA
Anti-Flag
TCL
c
IP: Anti-Flag
PP2
Flag-HK1
HA-c-Src
WB:Anti-HA
Anti-Flag
IP
Anti-HA
Anti-Flag
TCL
d
IP: Anti-Flag
Flag-HK1
HA-c-Src
HA-c-Src-KD
HA-c-Src-Y529F
WB: Anti-HA
1 0.41 1.61
Anti-Flag
IP
Anti-HA
Anti-Flag
TCL
e
Cox 8a-GFP
HK1-RFP
Flag-c-Src
DAPI
Merge
f
IP: Anti-HA
HA-c-Src
Flag-HK2
WB: Anti-Flag
Anti: HA
IP
Anti-Flag
Anti-HA
TCL
g
IP: Anti-Flag
HA-c-Src
Flag-HK2
WB: Anti-HA
Anti-Flag
IP
Anti-HA
Anti-Flag
TCL
h
Input
GST pull down
His-HK2
GST-c-Src
GST
130 100 70 55 40 35 25
WB:HK2
GST-c-Src
Truncated GST-c-Src
GST
i
IP: Anti-Flag
PP2
Flag-HK2
HA-c-Src
WB: Anti-Flag
Anti-HA
IP
Anti-Flag
Anti-HA
TCL
j
IP: Anti-Flag
Flag-HK2
HA-c-Src
HA-c-Src-KD
HA-c-Src-Y529F
WB: Anti-Flag
Anti-HA
1 0.45 1.50
IP
Anti-Flag
Anti-HA
TCL

Supplementary Figure 1. c-Src activity is essential for its interaction with HK1 and HK2. (**a**) SH2 (Src homology) domain is indispensable for c-Src to interact with HK1. Flag-HK1 was co-transfected into HEK 293T cells separately with HA-c-Src and its deletion mutants ΔSH3 (with aa 86-150 deleted), ΔSH2 (with aa 151-249 deleted) and Δ(SH3+SH2) (with aa 86-249 deleted). IPs were performed with M_2 beads (anti-Flag). Immunoprecipitates and total cell lysates were analyzed by WB with anti-HA for c-Src and anti-Flag for HK1. Myr, myristylation domain. (**b**) N-half (aa 1-454) of HK1 is mainly responsible for its interaction with c-Src. (**c**) PP2, a c-Src inhibitor, impaired the interaction between c-Src and HK1. HEK 293T cells were transfected with the combinations of plasmids as indicated. 24 hours posttransfection, cells were treated with 10 μM PP2 for 4 hours. The cell lysates were immunoprecipitated with M_2 beads. The immunoprecipitates were detected for proteins as depicted. (**d**) The activity of c-Src is essential for its interaction with HK1. c-Src, c-Src-KD and c-Src-Y529F was transfected alone, or together with Flag-HK1 into HEK 293T cells. Lysates were immunoprecipitated with M_2 beads, followed by detection with anti-Flag for HK1 and anti-HA for co-immunoprecipitated c-Src. (**e**) In HeLa cells, HK1 and c-Src complex showed less co-localization in mitochondria than in cytoplasm. Cox 8a was a mitochondrial marker. scale bar, 30μm (**f, g**) c-Src can interact with HK2. HEK 293T cells were transfected with HA-c-Src and Flag-HK2 in combinations as indicated. Reciprocal IPs were performed to precipitate HA-c-Src (**f**) and Flag-HK2 (**g**). (**h**) HK2 interacts with c-Src in vitro. GST pull down was carried out with bacterially expressed His-HK2 and GST-c-Src. GST protein was used as a negative control. Large amounts of His-HK2 were precipitated by GST-c-Src, but not by GST. Left panel, coomassie brilliant blue staining of precipitates; right panel, WB detection of the same precipitates. (**i**) The interaction between c-Src and HK2 was abrogated by treatment of HEK 293T cells with PP2. (**j**) c-Src kinase activity is required for its interaction with HK2 in HEK 293T cells.

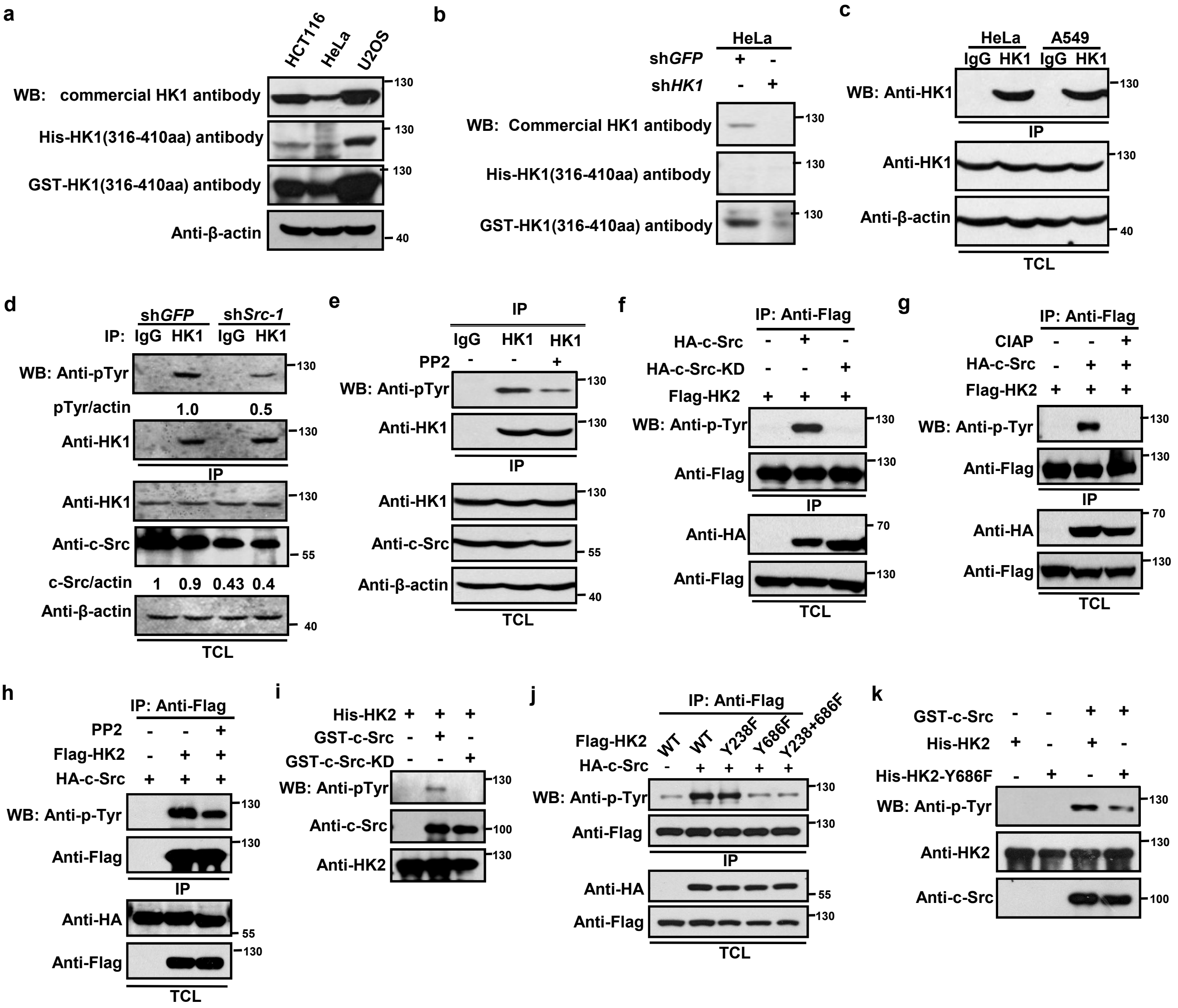

Supplementary Figure 2. Both HK1 and HK2 are phosphorylated by c-Src. (**a**) HK1 antibody raised with GST-HK1 (aa 316-410) as immunogen showed high valency towards HK1. Bacterially expressed GST- or His-tagged fragments (aa 316-410) of HK1 were employed to immunize rabbits, followed by affinity purification of corresponding antibodies. Purified antibodies were subjected to detect endogenous HK1 in HCT116, HeLa and U2OS cells. A commercially available HK1 antibody acts as a positive control. (**b**) High specificity of home-made anti-HK1 antibody is indicated by knock down of endogenous HK1 in HeLa. (**c**) Home-made HK1 antibody is suitable for immunoprecipitation. Endogenous HK1 in HeLa and A549 cells were successfully IPed by employing home-made anti-HK1 antibody. (**d**) Partial knockdown of *c-Src* in A549 cells diminishes tyrosine phosphorylation of endogenous HK1. (**e**) Tyrosine phosphorylation of endogenous HK1 was effectively abolished by treating A549 cells with PP2. (**f**) It is c-Src, but not c-Src-KD that strongly phosphorylates HK2 in HEK 293T cells. (**g**) Treatment of cell lysates with CIAP thoroughly abolished the tyrosine phosphorylation of HK2 mediated by c-Src in HEK 293T cells. (**h**) Phosphorylation level of HK2 induced by c-Src in HEK 293T cells was eliminated by the treatment of cells with PP2. (**i**) Recombinant HK2 can be effectively phosphorylated by bacterially purified c-Src rather than c-Src-KD. (**j**) The mutation of HK2 Y686F could abolish the tyrosine phosphorylation induced by c-Src in HEK 293T cells. (**k**) In vitro kinase assay showed that c-Src could phosphorylate HK2 WT but not HK2 Y686F.

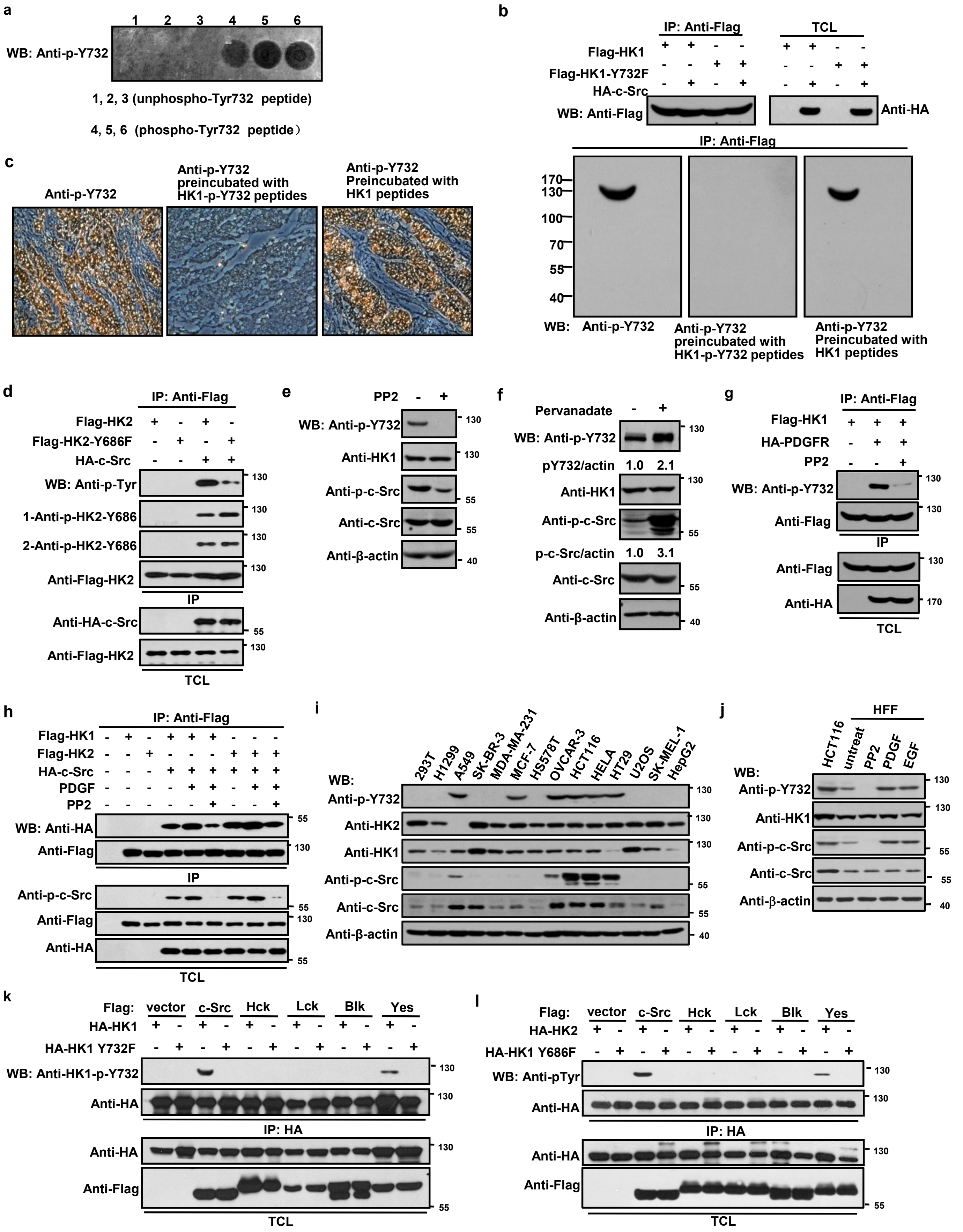

a
1 2 3 4 5 6
WB: Anti-p-Y732
1, 2, 3 (unphospho-Tyr732 peptide)
4, 5, 6 (phospho-Tyr732 peptide）
b
IP: Anti-Flag
TCL
Flag-HK1
Flag-HK1-Y732F
HA-c-Src
WB: Anti-Flag
Anti-HA
IP: Anti-Flag
WB: Anti-p-Y732
Anti-p-Y732 preincubated with HK1-p-Y732 peptides
Anti-p-Y732 Preincubated with HK1 peptides
c
Anti-p-Y732
Anti-p-Y732 preincubated with HK1-p-Y732 peptides
Anti-p-Y732 Preincubated with HK1 peptides
d
IP: Anti-Flag
Flag-HK2
Flag-HK2-Y686F
HA-c-Src
WB: Anti-p-Tyr
1-Anti-p-HK2-Y686
2-Anti-p-HK2-Y686
Anti-Flag-HK2
IP
Anti-HA-c-Src
Anti-Flag-HK2
TCL
e
PP2
WB: Anti-p-Y732
Anti-HK1
Anti-p-c-Src
Anti-c-Src
Anti-β-actin
f
Pervanadate
WB: Anti-p-Y732
pY732/actin 1.0 2.1
Anti-HK1
Anti-p-c-Src
p-c-Src/actin 1.0 3.1
Anti-c-Src
Anti-β-actin
g
IP: Anti-Flag
Flag-HK1
HA-PDGFR
PP2
WB: Anti-p-Y732
Anti-Flag
IP
Anti-Flag
Anti-HA
TCL
h
IP: Anti-Flag
Flag-HK1
Flag-HK2
HA-c-Src
PDGF
PP2
WB: Anti-HA
Anti-Flag
IP
Anti-p-c-Src
Anti-Flag
Anti-HA
TCL
i
293T H1299 A549 SK-BR-3 MDA-MA-231 MCF-7 HS578T OVCAR-3 HCT116 HELA HT29 U2OS SK-MEL-1 HepG2
WB:
Anti-p-Y732
Anti-HK2
Anti-HK1
Anti-p-c-Src
Anti-c-Src
Anti-β-actin
j
HFF
HCT116 untreat PP2 PDGF EGF
WB:
Anti-p-Y732
Anti-HK1
Anti-p-c-Src
Anti-c-Src
Anti-β-actin
k
Flag: vector c-Src Hck Lck Blk Yes
HA-HK1
HA-HK1 Y732F
WB: Anti-HK1-p-Y732
Anti-HA
IP: HA
Anti-HA
Anti-Flag
TCL
l
Flag: vector c-Src Hck Lck Blk Yes
HA-HK2
HA-HK1 Y686F
WB: Anti-pTyr
Anti-HA
IP: HA
Anti-HA
Anti-Flag
TCL

Supplementary Figure 3. c-Src phosphorylates HK1 at Tyr732 and HK2 at Tyr686. (**a**) The anti-p-Y732 antibody specifically recognized phosphorylated HK1. Keyhole limpet hemocyanin (KLH)-tagged oligo-peptides containing phosphorylated Y732 residue were used to immunize rabbits, followed by purification of anti-HK1-phospho-Y732 antibody (designated as anti-p-Y732) with CNBr beads. The specificity of anti-p-Y732 was determined by detecting KLH-tagged peptides with or without Y732 phosphorylated by immunodot blotting. (**b**) The specificity of our self-made anti-p-Y732 antibody was confirmed by WB with this antibody preincubated with HK1-p-Y732 or HK1 peptides. The expression plasmids encoding HK1 and HK1 Y732F were transfected into HEK 293T cells with or without HA-c-Src. Cell lysates were used to immunoprecipitation by M_2 beads and followed by WB with indicated antibodies. (**c**) The antibody against to HK1 Y732 phosphorylation is specific for immunohistochemistry. Colorectal tissues were immunostainned with the corresponding antibodies. (**d**) Two antibodies prepared by using synthesized HK2 peptides containing phosphorylated Y686 residue as immunogen failed to specifically recognize phosphorylated HK2. 1 and 2 refer to serums from two immunized rabbits. (**e**) Inhibition of c-Src activity by PP2 resulted in decreasing HK1-Y732 phosphorylation. (**f**) HK1-Y732 phosphorylation was significantly increased by exposure of A549 cells to 0.1 mM pervanadate for 10 minutes. (**g**) Treatment with PP2 could block the HK1 Y732 phosphorylation induced by overexpression of PDGFR in HEK 293T cells. (**h**) The associations between HK 1/HK2 and c-Src were enhanced by stimulating cells with PDGF, but this effect could be reversed by co-treatment with PP2. After 24 hours of transfection, HEK 293T cells were exposed to PP2 4 hours and PDGF for 10 minutes alone or in combination. (**i**) Y732 phosphorylation levels of HK1 strongly correlate with Y419 phosphorylation levels of c-Src in various cancer cell lines. (**j**) Both PDGF (20 ng ml^{-1}) and EGF (100 ng ml^{-1}) effectively stimulated HK1-Y732 phosphorylation in human foreskin fibroblast (HFF) cells. (**k, l**) Among four Src family members examined other than c-Src, Yes showed much weak effect on HK1 Y732 phosphorylation (**k**) and HK2 tyrosine phosphorylation (**l**) in HEK 293T cells compared to c-Src.

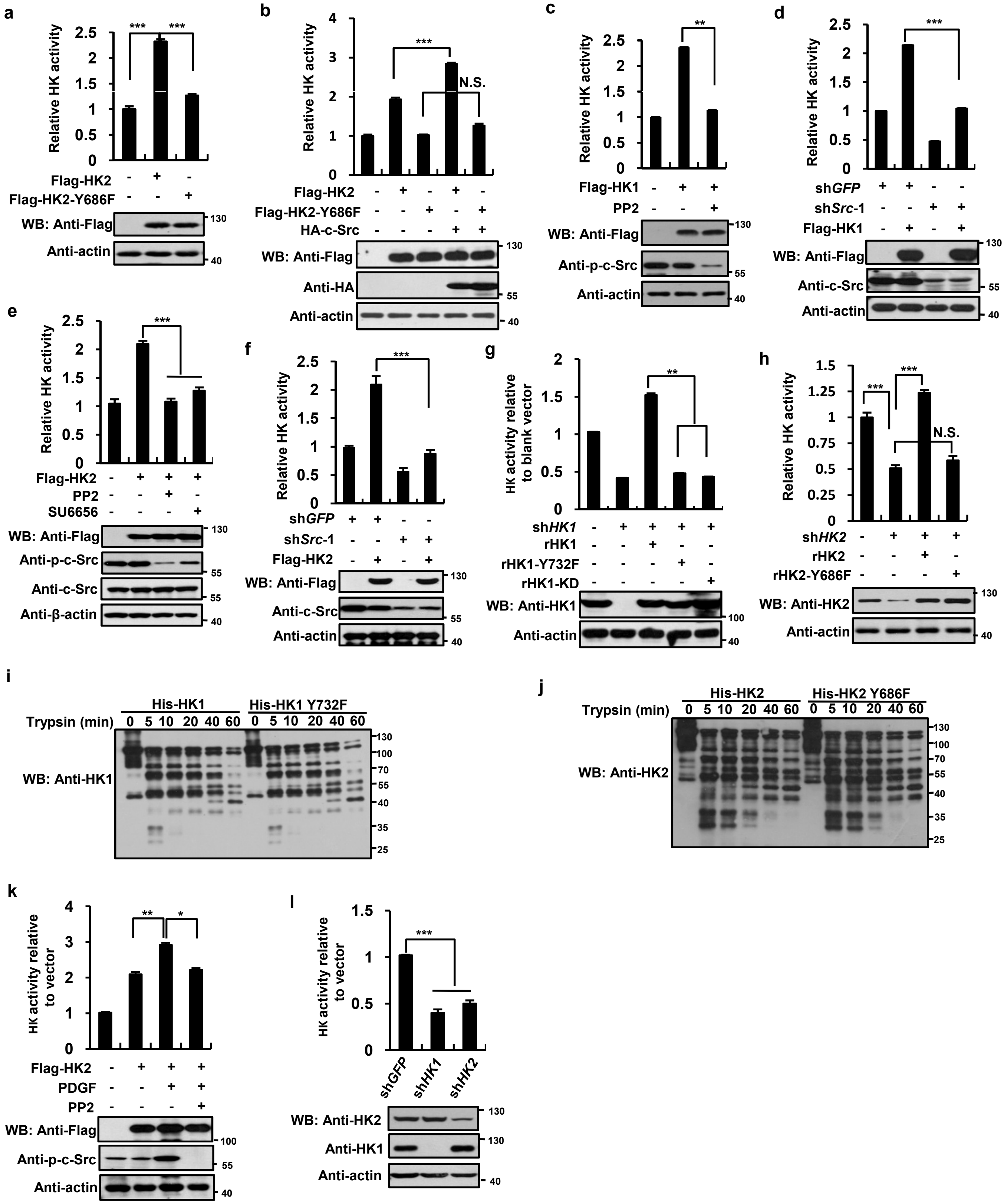

a
Relative HK activity
Flag-HK2
Flag-HK2-Y686F
WB: Anti-Flag
Anti-actin
b
Relative HK activity
N.S.
Flag-HK2
Flag-HK2-Y686F
HA-c-Src
WB: Anti-Flag
Anti-HA
Anti-actin
c
Relative HK activity
Flag-HK1
PP2
WB: Anti-Flag
Anti-p-c-Src
Anti-actin
d
Relative HK activity
shGFP
shSrc-1
Flag-HK1
WB: Anti-Flag
Anti-c-Src
Anti-actin
e
Relative HK activity
Flag-HK2
PP2
SU6656
WB: Anti-Flag
Anti-p-c-Src
Anti-c-Src
Anti-β-actin
f
Relative HK activity
shGFP
shSrc-1
Flag-HK2
WB: Anti-Flag
Anti-c-Src
Anti-actin
g
HK activity relative to blank vector
shHK1
rHK1
rHK1-Y732F
rHK1-KD
WB: Anti-HK1
Anti-actin
h
Relative HK activity
N.S.
shHK2
rHK2
rHK2-Y686F
WB: Anti-HK2
Anti-actin
i
His-HK1
His-HK1 Y732F
Trypsin (min)
0 5 10 20 40 60 0 5 10 20 40 60
WB: Anti-HK1
j
His-HK2
His-HK2 Y686F
Trypsin (min)
0 5 10 20 40 60 0 5 10 20 40 60
WB: Anti-HK2
k
HK activity relative to vector
Flag-HK2
PDGF
PP2
WB: Anti-Flag
Anti-p-c-Src
Anti-actin
l
HK activity relative to vector
shGFP
shHK1
shHK2
WB: Anti-HK2
Anti-HK1
Anti-actin

Supplementary Figure 4. c-Src promotes catalytic activities of both HK1 and HK2. (**a**) In HEK 293T cells, HK2-Y686F showed greatly decreased enzymatic activity as compared to WT HK2. (**b**) Co-transfection of c-Src enhanced the activity of HK2, but not HK2-Y686F in HEK 293T cells. (**c**) PP2 effectively inhibited HK1 activity. A549 was overexpressed with Flag-HK1 followed by treatment with or without PP2. Hexokinase activity was determined as described in Methods. (**d**) Knock-down of c-Src diminished catalytic ability of HK1. Flag-HK1 was expressed in A549 cells with c-Src already knocked-down. HK activity was measured accordingly. (**e**) Treatment with PP2 or SU6656 disrupted the increase of hexokinase activity induced by overexpression of HK2 in HeLa cells. (**f**) Knock down of c-Src abolished the augment of hexokinase activity caused by expression of Flag-HK2 in HeLa cells. (**g**) Re-expression of HK1, but not HK1-Y732F or HK1-KD in HK1 knocked-down A549 cells rescued hexokinase activity. (**h**) Re-expressed HK2, but not its mutation, rescued the HK activity of HeLa cells deprived of endogenous HK2. (**i, j**) The digestion patterns of HK mutants were the same as their wild type controls. Equal amounts of HK and their mutant protein were digested with trypsin (200 μM) at 37 ℃ for indicated times, followed by SDS-PADE and Western blot to determine the digestion patterns. (**k**) PDGF functioned to stimulate HK2 enzyme activity, and such effect was neutralized by treatment of cells with PP2 in HeLa cells. (**l**) HK1 and HK2 were knocked down with corresponding shRNAs, followed by measurement of total HK activity accordingly. After normalization of enzyme activities to corresponding knockdown efficiencies, two isozymes contribute almost equally to total HK activity in HeLa cells. HK activities in each panel were normalized to the control cells (the first bar) and shown as means±s.d. (three experimental replicates). Unpaired student's *t* test was used to analyze the significance. *$p<0.05$, **$p<0.01$, ***$p<0.001$, N.S. represents no significant difference.

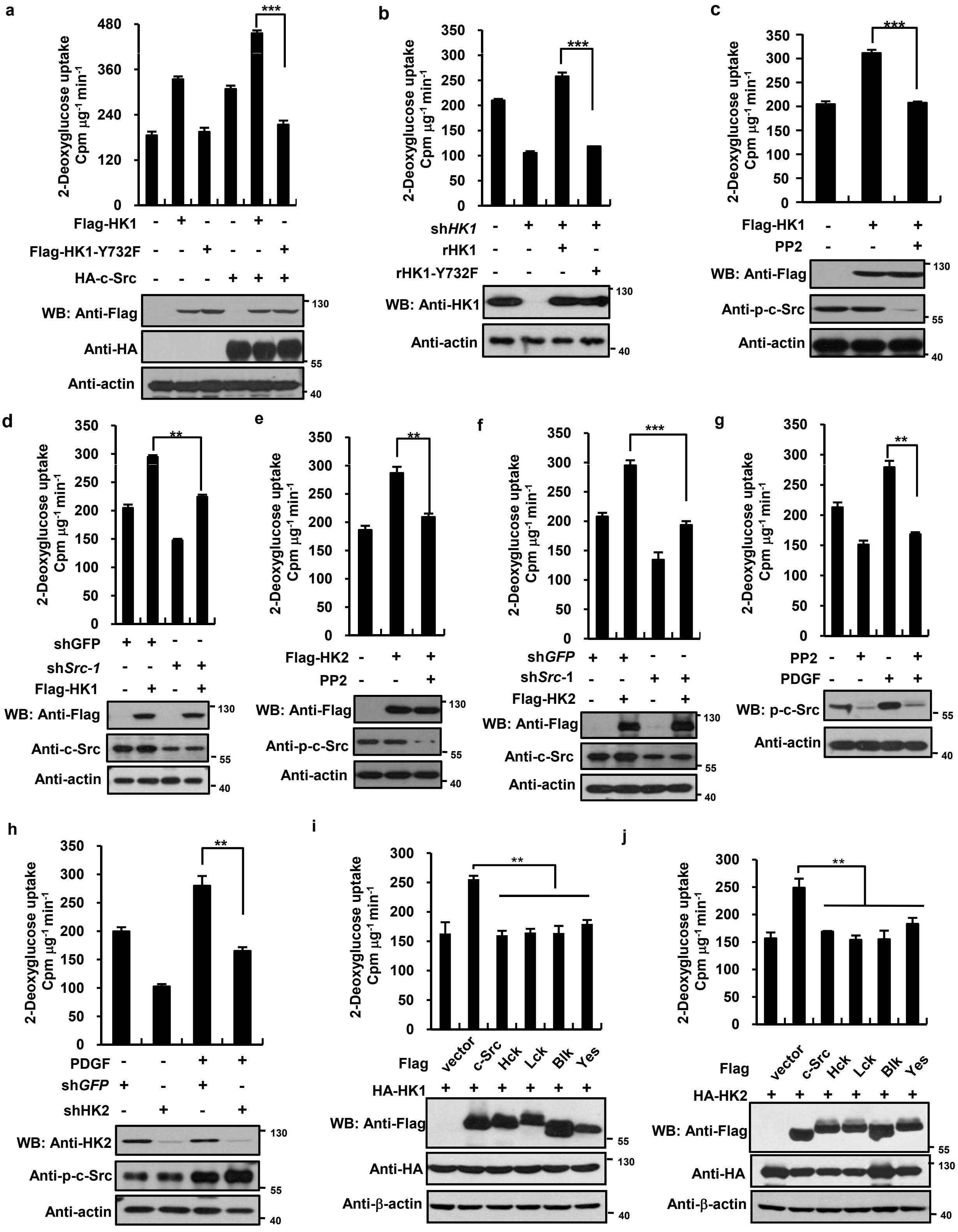
a
b
c
d
e
f
g
h
i
j
2-Deoxyglucose uptake
Cpm µg-1 min-1
***
**
Flag-HK1
Flag-HK1-Y732F
HA-c-Src
WB: Anti-Flag
Anti-HA
Anti-actin
shHK1
rHK1
rHK1-Y732F
WB: Anti-HK1
PP2
Anti-p-c-Src
shGFP
shSrc-1
Anti-c-Src
Flag-HK2
PDGF
WB: p-c-Src
shHK2
WB: Anti-HK2
Flag
vector
c-Src
Hck
Lck
Blk
Yes
HA-HK1
HA-HK2
Anti-β-actin
130
55
40

Supplementary Figure 5. HK1/HK2-catalyzed glycolysis is stimulated by c-Src. (**a**) HK1-promoted glucose uptake was enhanced by overexpression of c-Src. A549 cells were infected with lentiviruses expressing the proteins indicated. HK1-Y732F was used as a negative control. (**b**) Introduction of WT HK1, but not its Y732F mutant, rescued the attenuation of hexokinase activity caused by knock-down of endogenous HK1 in A549 cells. (**c**) HK1-stimulated glucose uptake was abolished by exposure of A549 cells to PP2. (**d**) Glucose uptake stimulated by overexpression of HK1 was abrogated by knock-down of c-Src in A549 cells. (**e**) HK2-mediated glucose uptake was suppressed by PP2 in HeLa cells. (**f**) The stimulation of glycolysis by HK2 in HeLa cells was attenuated by knock-down of c-Src. (**g**) PDGF-promoted glucose uptake was inhibited by exposure of A549 cells to PP2. (**h**) PDGF stimulated glucose uptake in HeLa cells was diminished by knock-down of HK2. (**i, j**) Other four Src family members examined showed much weaker effect on HK1 (**i**) and HK2 (**j**) promoted glucose uptake compared to c-Src. Results of each panel are means±s.d. (three experimental replicates). Unpaired student's *t* test was used to analyze the significance. **$p<0.01$, ***$p<0.001$.

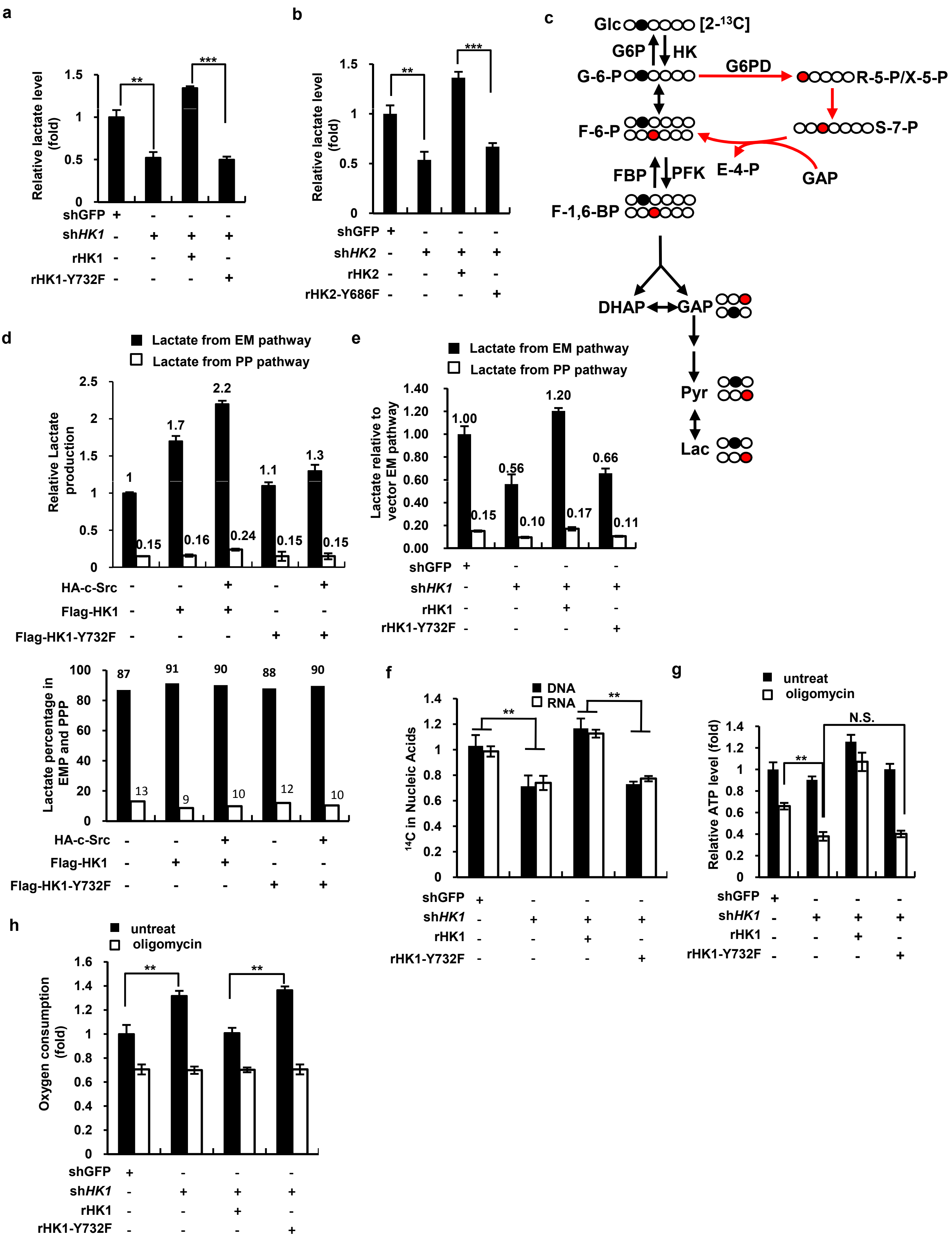
a
Relative lactate level (fold)
1.5
1
0.5
0
**
***
shGFP + - - -
shHK1 - + + +
rHK1 - - + -
rHK1-Y732F - - - +
b
Relative lactate level (fold)
1.5
1
0.5
0
**
***
shGFP + - - -
shHK2 - + + +
rHK2 - - + -
rHK2-Y686F - - - +
c
Glc [2-13C]
G6P
HK
G-6-P
G6PD
R-5-P/X-5-P
F-6-P
S-7-P
FBP
PFK
E-4-P
GAP
F-1,6-BP
DHAP
GAP
Pyr
Lac
d
Lactate from EM pathway
Lactate from PP pathway
Relative Lactate production
2.5
2
1.5
1
0.5
0
1
0.15
1.7
0.16
2.2
0.24
1.1
0.15
1.3
0.15
HA-c-Src - - + - +
Flag-HK1 - + + - -
Flag-HK1-Y732F - - - + +
Lactate percentage in EMP and PPP
100
80
60
40
20
0
87
13
91
9
90
10
88
12
90
10
HA-c-Src - - + - +
Flag-HK1 - + + - -
Flag-HK1-Y732F - - - + +
e
Lactate from EM pathway
Lactate from PP pathway
Lactate relative to vector EM pathway
1.40
1.20
1.00
0.80
0.60
0.40
0.20
0.00
1.00
0.15
0.56
0.10
1.20
0.17
0.66
0.11
shGFP + - - -
shHK1 - + + +
rHK1 - - + -
rHK1-Y732F - - - +
f
DNA
RNA
14C in Nucleic Acids
1.4
1.2
1
0.8
0.6
0.4
0.2
0
**
**
shGFP + - - -
shHK1 - + + +
rHK1 - - + -
rHK1-Y732F - - - +
g
untreat
oligomycin
Relative ATP level (fold)
1.4
1.2
1
0.8
0.6
0.4
0.2
0
**
N.S.
shGFP + - - -
shHK1 - + + +
rHK1 - - + -
rHK1-Y732F - - - +
h
untreat
oligomycin
Oxygen consumption (fold)
1.6
1.4
1.2
1
0.8
0.6
0.4
0.2
0
**
**
shGFP + - - -
shHK1 - + + +
rHK1 - - + -
rHK1-Y732F - - - +

Supplementary Figure 6. c-Src augments glucose flux through both EMP and PPP. (**a**, **b**) Re-expression of wildtype HK, but not its mutant, rescued the lactate production in HeLa cells. (**c**) A schematic diagram indicates [2-^{13}C] glucose metabolic fates through EMP and PPP. ^{13}C atom is drawn as filled circles. The carbon atoms going through the EMP are labeled as black filled circles. The circles in red represent the ^{13}C atoms going through PPP and then recycled back to EMP. (**d**) Overexpressed HK1 rather than HK1-Y732F efficiently increased lactate secretion through both EMP and PPP, and such an effect was enhanced by co-expression of c-Src (upper panel). The corresponding percentages of glucose metabolized through EMP and PPP are shown in lower panel. HK1, HK1-Y732F and c-Src were expressed by infecting HeLa cells with different combinations of lentiviruses as indicated. Lactate secretion was measured by NMR. (**e**) In HK1 knock down HeLa cells, the lactate production ratio from both EMP and PPP was downregulated and this effect was reversed by re-expression of WT HK1, but not its mutant. (**f**) Disruption of HK1 expression by shRNA influenced DNA/RNA synthesis. HeLa cells were treated with 6-^{14}C glucose for 24 hours, the incorporation ratio were determined according to the protocol in Methods. (**g**) Oligomycin treatment resulted in enhanced inhibition of ATP synthesis in HK1 knock down and HK1-Y732F rescue HeLa cells compared with sh*GFP* cells. (**h**) Rescue expression of HK1, but not its mutant, reversed the increase of oxygen consumption caused by interference of HK1. All the results represent means±s.d. of three independent experiments. Unpaired Student's *t* test was used to analyze the significance. $^{**}p<0.01$, $^{***}p<0.001$,.

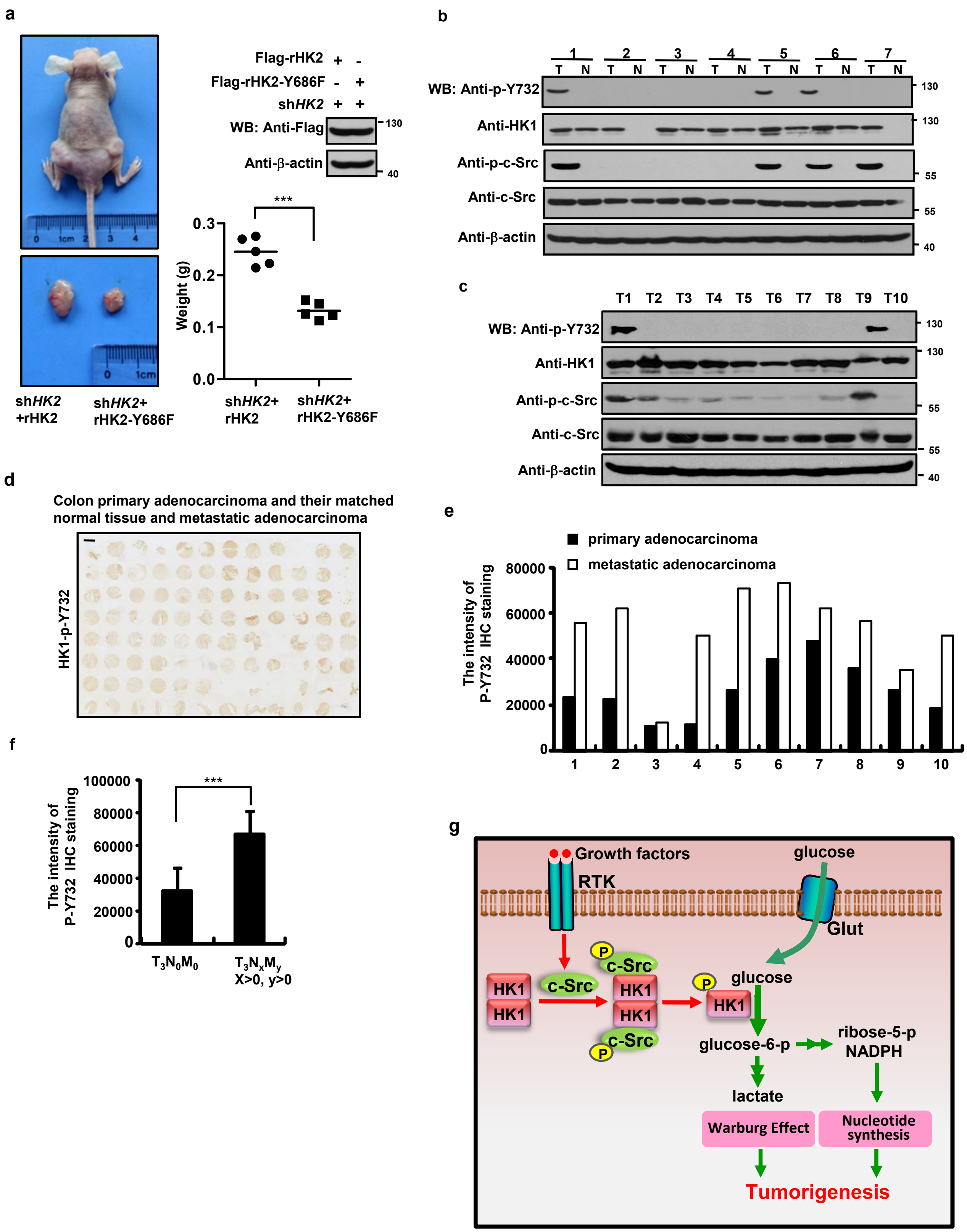
a
Flag-rHK2 + -
Flag-rHK2-Y686F - +
shHK2 + +
WB: Anti-Flag
Anti-β-actin
130
40
***
0.3
0.2
0.1
0.0
Weight (g)
shHK2+rHK2
shHK2+rHK2-Y686F
shHK2 +rHK2
shHK2+ rHK2-Y686F
b
1 2 3 4 5 6 7
T N T N T N T N T N T N T N
WB: Anti-p-Y732
Anti-HK1
Anti-p-c-Src
Anti-c-Src
Anti-β-actin
130
130
55
55
40
c
T1 T2 T3 T4 T5 T6 T7 T8 T9 T10
WB: Anti-p-Y732
Anti-HK1
Anti-p-c-Src
Anti-c-Src
Anti-β-actin
130
130
55
55
40
d
Colon primary adenocarcinoma and their matched normal tissue and metastatic adenocarcinoma
HK1-p-Y732
e
primary adenocarcinoma
metastatic adenocarcinoma
The intensity of P-Y732 IHC staining
80000
60000
40000
20000
0
1 2 3 4 5 6 7 8 9 10
f
The intensity of P-Y732 IHC staining
100000
80000
60000
40000
20000
0
***
$T_3N_0M_0$
$T_3N_xM_y$
X>0, y>0
g
Growth factors
RTK
glucose
Glut
P
c-Src
HK1
HK1
HK1
HK1
c-Src
P
P
HK1
glucose
glucose-6-p
ribose-5-p
NADPH
lactate
Warburg Effect
Nucleotide synthesis
Tumorigenesis

Supplementary Figure 7. Phosphorylation of HK1-Y732 is significantly correlated with c-Src activity and tumor metastasis. (**a**) HK2-Y686F impeded the xenograft tumor formation relative to WT HK2. The tumor lysates were detected with WB as indicated. The data were analyzed by employing two-way ANOVA (***p<0.001). (**b**) The activity of c-Src is positively correlated to the phosphorylation of HK1. The tumor lysates from patients suffered from breast cancer were detected with antibodies indicated. (**c**) Glioma lysates were immunoblotted with the antibodies indicated. (**d**) IHC staining of a microarray containing primary colon adenocarcinomas and their matched normal tissues and metastatic adenocarcinomas with anti-p-Y732 antibody. The scale bar is 1.5 mm. (**e**) The phosphorylation intensity of HK1 Y732 is much higher in metastatic colon adenocarcinoma tissues than in primary colon adenocarcinoma tissues. The slides quantifications were carried out with IPP software. Unpaired Student's t test was used to analyze the significance (p<0.001, n=10). (**f**) The phosphorylation intensity of HK1 Y732 is much higher in primary colon adenocarcinoma tissues with lymph node or distant metastasis than in primary colon adenocarcinoma tissues without any metastasis. Unpaired Student's t test was used to analyze the significance (***p<0.001, n=7). This statistical result is from the IHC staining shown in Supplementary Fig. 7d. (**g**) A schematic diagram showing the mechanism how c-Src activates HK1. Upon the stimulation of growth factors, such as EGF, c-Src is activated to form a transient protein complex with HK1 homo-dimer. In this complex, HK1 is promptly phosphorylated by c-Src at Y732, which is essential for rapid liberation of HK1 from the complex. Librated HK1 exists in the form of phosphorylated monomer which shows maximum catalytic efficiency. After activation, HK1 increases glucose metabolism through both EMP and PPP, which in turn provides enough ATP, ribose-5-P, NADPH and other intermediates required for biosynthesis of rapidly proliferating tumor cells.

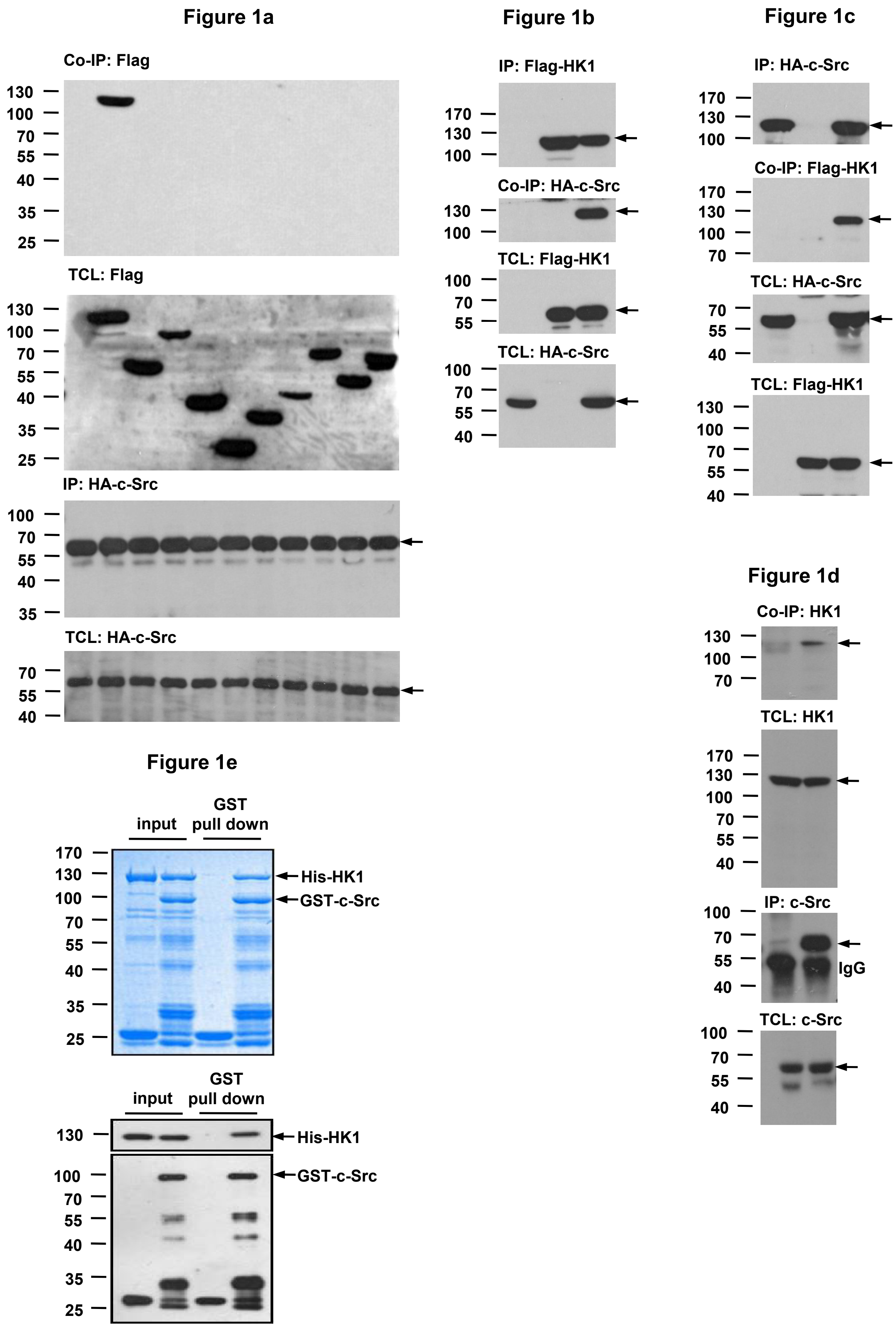

Supplementary Figure 8. Uncropped images of western blots analysis (Figure 1)

Figure 2a

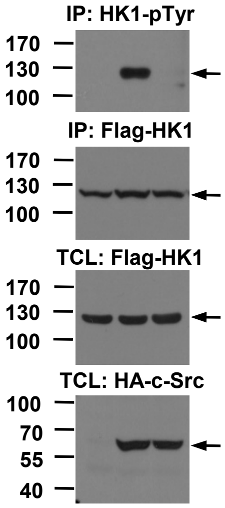

Figure 2b

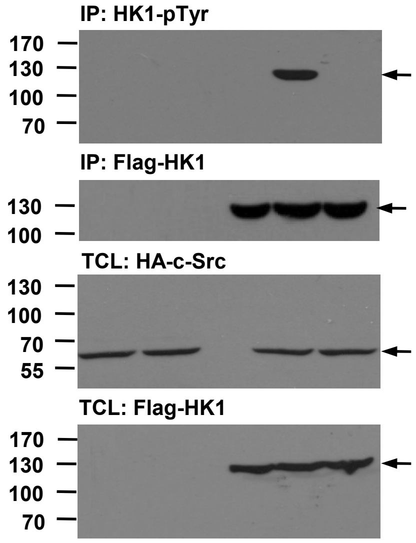

Figure 2c

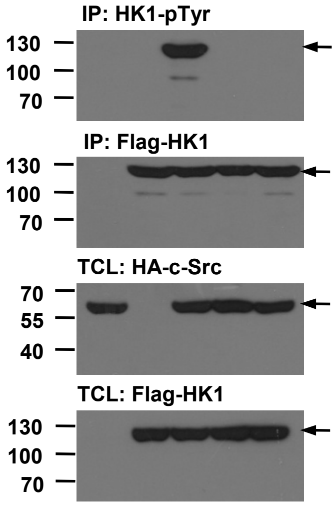

Figure 2d

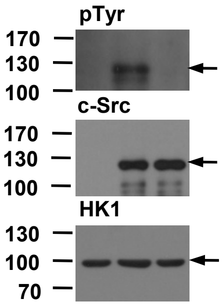

Figure 2e

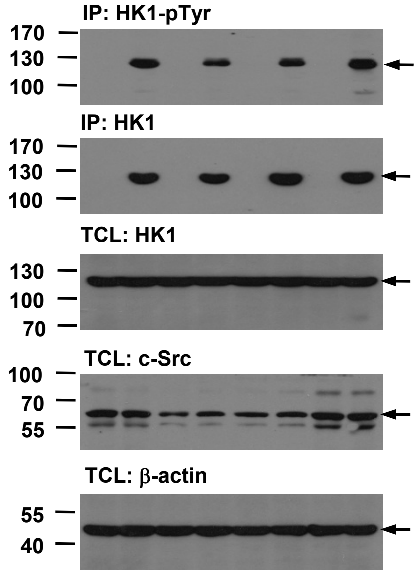

Figure 2f

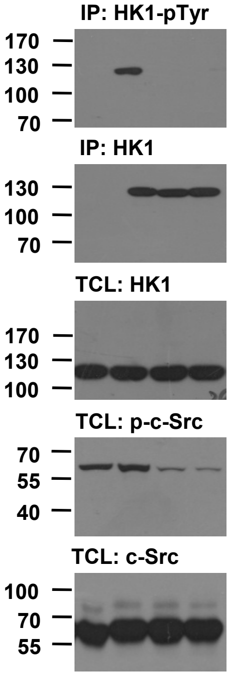

Supplementary Figure 9. Uncropped images of western blots analysis (Figure 2) (continue)

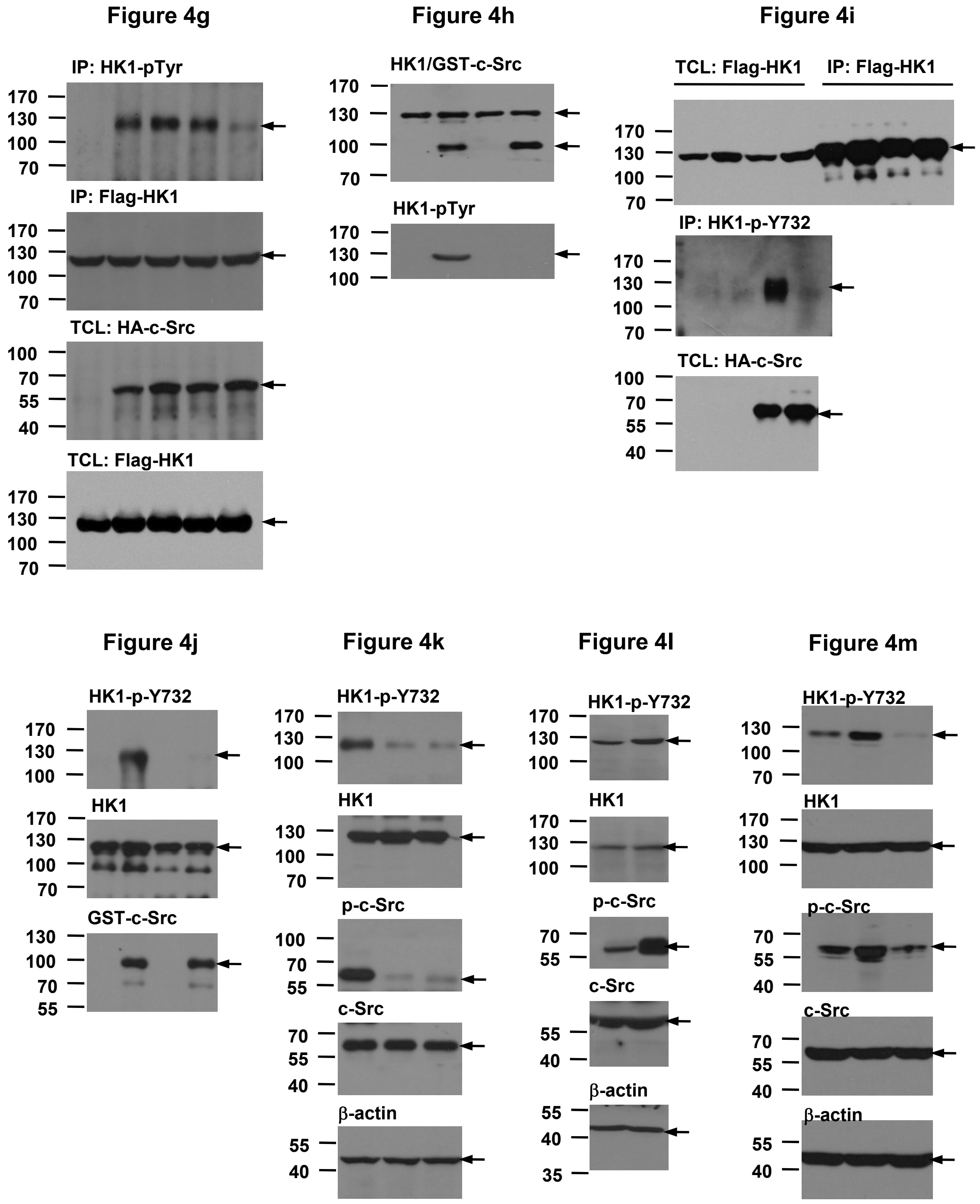

Supplementary Figure 9. Uncropped images of western blots analysis (Figure 2)

Figure 4c

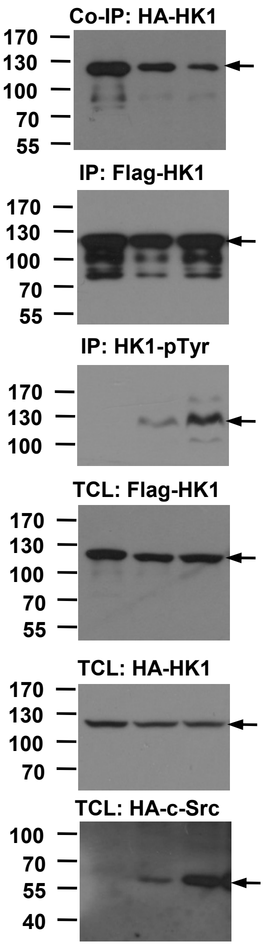

Figure 4d

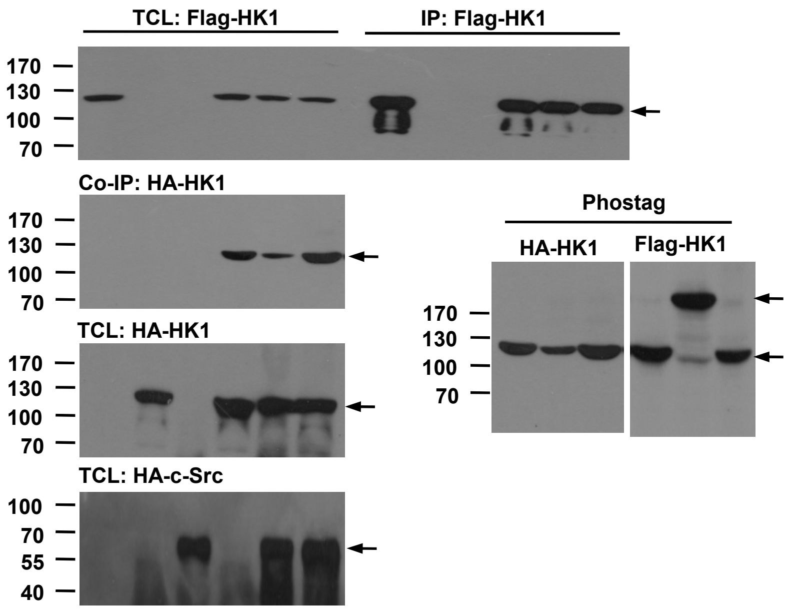

Figure 4e

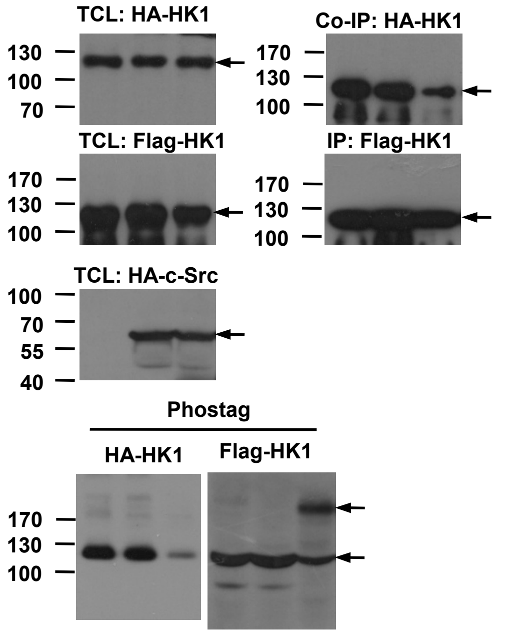

Figure 4g

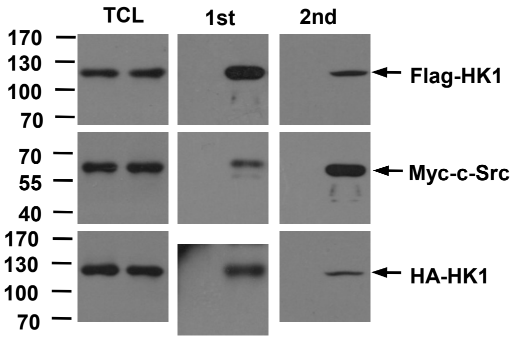

Supplementary Figure 10. Uncropped images of western blots analysis (Figure 4)

Figure 8c

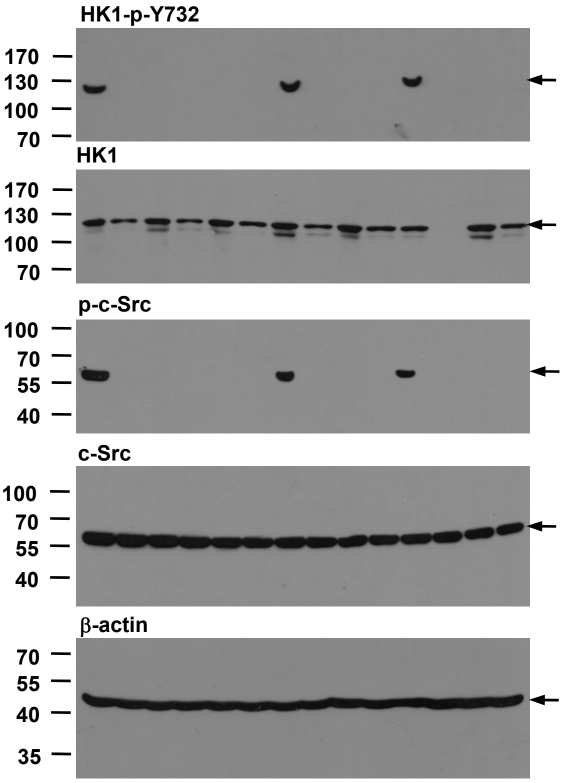

Figure 8d

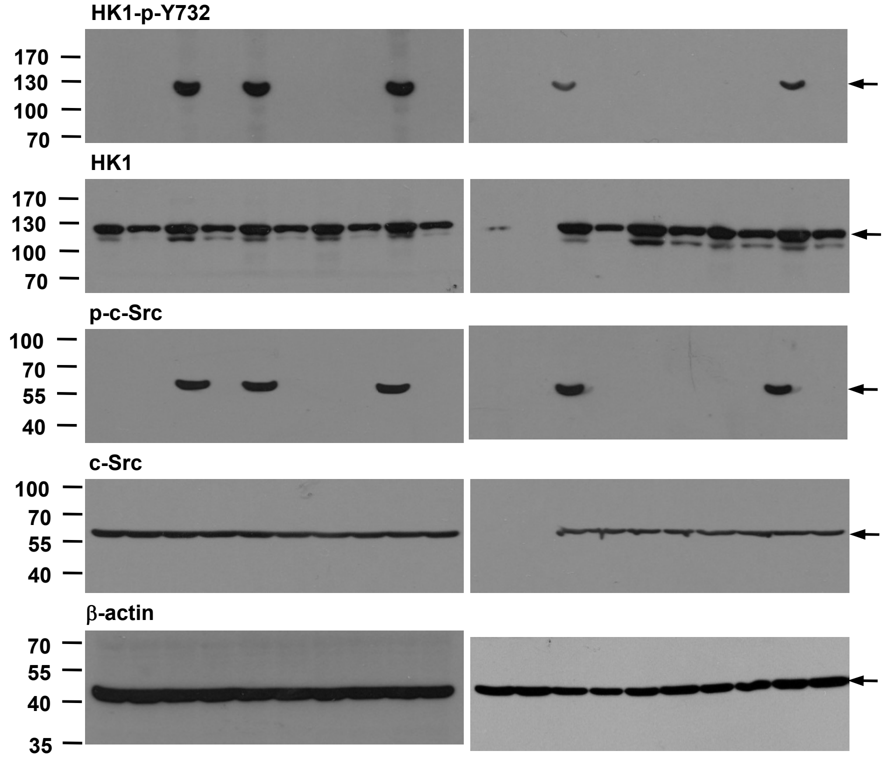

Supplementary Figure 11. Uncropped images of western blots analysis (Figure 8)
